# Targeting senescence induced by age or chemotherapy with a polyphenol-rich natural extract improves longevity and healthspan in mice

**DOI:** 10.1038/s43587-024-00663-7

**Published:** 2024-07-01

**Authors:** Sara Zumerle, Miles Sarill, Miriam Saponaro, Manuel Colucci, Liliana Contu, Edoardo Lazzarini, Roberta Sartori, Camilla Pezzini, Anna Rinaldi, Anna Scanu, Jacopo Sgrignani, Patrizia Locatelli, Marianna Sabbadin, Aurora Valdata, Daniela Brina, Isabella Giacomini, Beatrice Rizzo, Alessandra Pierantoni, Saman Sharifi, Silvia Bressan, Claudia Altomare, Yulia Goshovska, Chiara Giraudo, Roberto Luisetto, Luca Iaccarino, Cristina Torcasio, Simone Mosole, Emiliano Pasquini, Andrea Rinaldi, Laura Pellegrini, Gregorio Peron, Matteo Fassan, Stefano Masiero, Andrea Maria Giori, Stefano Dall’Acqua, Johan Auwerx, Pietro Cippà, Andrea Cavalli, Marco Bolis, Marco Sandri, Lucio Barile, Monica Montopoli, Andrea Alimonti

**Affiliations:** 1 Veneto Institute of Molecular Medicine, Padova, Italy; 2 Department of Medicine, University of Padova, Padova, Italy; 3 Department of Pharmaceutical and Pharmacological Sciences, University of Padova, Padova, Italy; 4 Department of Urology and Pediatric Urology, University Medical Center Bonn (UKB), Bonn, Germany; 5 Institute of Oncology Research (IOR), Bellinzona, Switzerland; 6 Università della Svizzera italiana, Lugano, Switzerland; 7 Faculty of Biology and Medicine, University of Lausanne UNIL, Lausanne, Switzerland; 8 Cardiovascular Theranostics, Istituto Cardiocentro Ticino, Laboratories for Translational Research, Ente Ospedaliero Cantonale, Bellinzona, Switzerland; 9 Faculty of Biomedical Sciences, Euler Institute, Università della Svizzera Italiana, Lugano, Switzerland; 10 Department of Biomedical Sciences, University of Padova, Padova, Italy; 11 Department of Medicine, Division of Nephrology, Ente Ospedaliero Cantonale, Lugano, Switzerland; 12 Laboratories for Translational Research, Ente Ospedaliero Cantonale, Bellinzona, Switzerland; 13 Department of Neuroscience, Rehabilitation Unit, University of Padova, Padova, Italy; 14 Institute for Research in Biomedicine, Università della Svizzera italiana, Bellinzona, Switzerland; 15 Veneto Institute of Oncology IOV – IRCCS, Padova, Italy; 16 Department of Health Sciences and Technology (D-HEST), ETH Zurich, Zurich, Switzerland; 17 Institute for Research on Cancer and Aging, Nice, France; 18 Department of Cardiac, Thoracic, Vascular Sciences and Public Health – DCTV, University of Padova, Padova, Italy; 19 Department of Surgery, Oncology and Gastroenterology, University of Padova, Padova, Italy; 20 Department of Molecular and Translational Medicine, University of Brescia, Brescia, Italy; 21 R&D Department, IBSA Farmaceutici Italia, Lodi, Italy; 22 Laboratory of Integrative Systems Physiology, Institute of Bioengineering, École Polytechnique Fédérale de Lausanne, Lausanne, Switzerland; 23 Oncology Institute of Southern Switzerland, Ente Ospedaliero Cantonale, Bellinzona, Switzerland

**Keywords:** Senescence, Ageing, Mechanisms of disease

## Abstract

Accumulating senescent cells within tissues contribute to the progression of aging and age-related diseases. Botanical extracts, rich in phytoconstituents, present a useful resource for discovering therapies that could target senescence and thus improve healthspan. Here, we show that daily oral administration of a standardized extract of *Salvia haenkei* (Haenkenium (HK)) extended lifespan and healthspan of naturally aged mice. HK treatment inhibited age-induced inflammation, fibrosis and senescence markers across several tissues, as well as increased muscle strength and fur thickness compared with age-matched controls. We also found that HK treatment reduced acutely induced senescence by the chemotherapeutic agent doxorubicin, using p16^LUC^ reporter mice. We profiled the constituent components of HK by mass spectrometry, and identified luteolin—the most concentrated flavonoid in HK—as a senomorphic compound. Mechanistically, by performing surface plasmon resonance and in situ proximity ligation assay, we found that luteolin disrupted the p16–CDK6 interaction. This work demonstrates that administration of HK promotes longevity in mice, possibly by modulating cellular senescence and by disrupting the p16–CDK6 interaction.

## Abstract (web summary)

Botanical extracts offer a valuable resource for identifying therapies. Zumerle, Sarill et al. show that a standardized extract of *Salvia haenkei* extends lifespan and healthspan in naturally aged mice by modulating inflammation and cellular senescence, and identify the constituent component luteolin as a senomorphic that disrupts the p16–CDK6 interaction.

## Main

In high-income countries, increased life expectancy and reduced birthrate have led to an aging population^1^. Aging is associated with a rise in age-related disorders such as cancer, cardiovascular, metabolic, kidney and neurodegenerative diseases, which represent the leading causes of death worldwide^2–4^. This results in a reduced quality of life for older adults, and increased healthcare spending, thus representing both a societal and economic burden^5^. Therefore, current research in the field of aging focuses on improving healthspan (that is, the number of years a person is in good health) rather than lifespan^2^. It is now widely accepted that treating aging as a whole, rather than single pathologies, may represent a more impactful strategy to tackle age-related disorders and increase the healthspan of older adults^6^. The accumulation of senescent cells within the body is a main contributor to organismal aging^7,8^. Cellular senescence, defined as a stable arrest of cell division, can be triggered by exogenous cellular insults such as DNA-damaging agents, ultraviolet (UV) irradiation or endogenous cellular stressors such as age-associated telomere attrition or the expression of oncogenes. Senescence is characterized by an upregulation of cell cycle inhibitors such as p16^INK4a^ (p16), which disrupt the G_1_ to S phase transition of the cell cycle^9^. p16 inhibits the kinase activity of CDK4 and CDK6, preventing the hyperphosphorylation of Rb and the release of E2F^9^. Although senescent cells are incapable of cell cycle progression, their accumulation within tissues contributes to age-related degeneration through the secretion of several inflammatory factors known together as the senescence-associated secretory phenotype (SASP)^10^. Disruption of the tissue microenvironment by SASP release can accelerate aging by driving senescent cell accumulation in a paracrine and systemic manner^4^.

It has been demonstrated that the selective removal of senescent cells can increase lifespan and restore the healthspan of aged animals^11–15^. Botanical extracts have long been used to treat age-related diseases in indigenous, traditional or folk medicines^16^. An expanding body of research has identified phytochemical constituents (for example, polyphenols, flavonoids, terpenoids) with putative anti-aging properties^17,18^. Plant-derived nutrients have been described to prolong healthspan through mechanisms such as the Nrf2-mediated induction of antioxidant defense, mitophagy activation, sirtuin induction or mTOR and protein translation inhibition, among others^19–23^.

Using high-throughput screening of 3,065 botanical, marine and synthetic compounds, we previously identified an extract of the Bolivian prawn sage (*Salvia haenkei* (SH)) that delays senescence in vitro^24^. SH significantly reduced the number of positive senescence-associated-β-galactosidase (SA-β-Gal) cells using different in vitro murine and human models^24^. Whereas senolytic compounds selectively kill senescent cells to promote an improved organismal healthspan, SH displayed senomorphic properties. Senomorphic compounds (also referred to as senostatics) modulate the function and morphology of senescent cells, restoring functionality to levels similar to those of young cells or delaying the accumulation of senescent cells in tissues^25^. SH has also been shown to promote wound healing, prevent formation of reactive oxygen species (ROS) and delay common aging phenotypes in HaCaT cells by upregulating several factors, such as occludin, filaggrin, and SIRT1 (ref. ^26^).

In the present study, we test the in vivo anti-aging potential of a standardized extract of SH*,* referred to as Haenkenium (HK). We report that treating naturally aged mice with a daily low dose of HK in drinking water decreased the accumulation of senescent cells and alleviated several aging-related parameters, including lifespan, physical fitness, fibrosis, bone mineralization and inflammation. HK treatment also decreased senescence in mice with therapy-induced senescence (TIS). We characterized the chemical composition of HK and identified the dietary flavonoid luteolin and luteolin-7-O-glucuronide as putative active compounds in the HK phytocomplex. Luteolin has previously been identified in the literature to promote antioxidant and anti-inflammatory effects in different models^21,27^. In the present study, we thus propose a mechanism by which the flavonoid luteolin enables senescence inhibition through disruption of the p16–CDK6 interaction, which may have applicability for the development of future therapeutics.

## Results

### HK expands healthspan and lifespan in mice

**Fig. 1:**
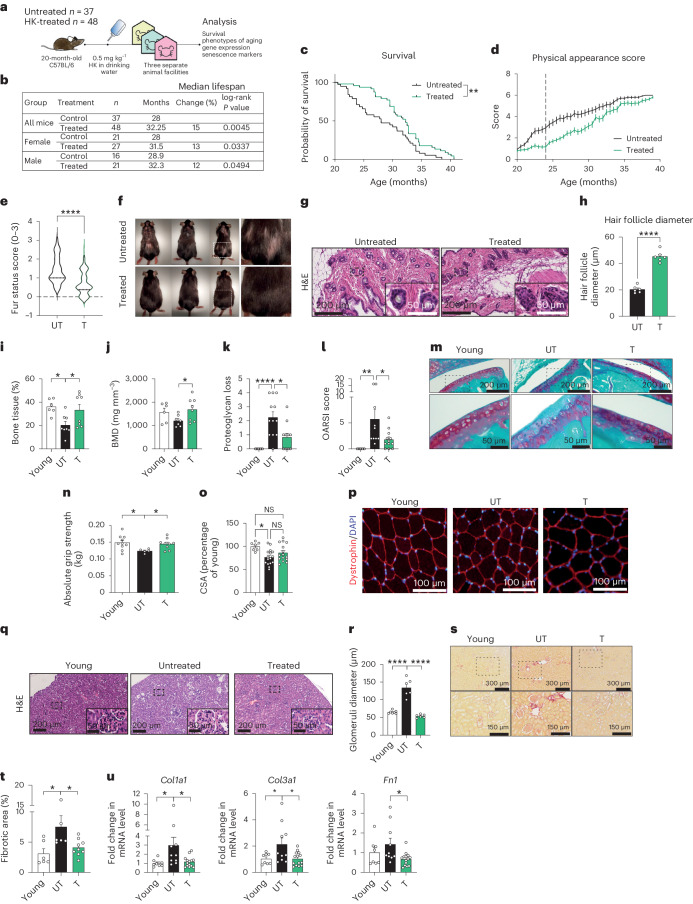
HK expands healthspan and lifespan in mice. **a**, Schematic representation of the experimental design. **b**, Lifespan analysis of treated and untreated mice; *n* = number of mice tested in each group; the percentage change was calculated with respect to control untreated mice. **c**, Survival curves for untreated (*n* = 37) and treated (*n* = 48) mice. **d**, Physical appearance score progression in untreated (UT) and treated (T) animals (UT *n* = 37; T *n* = 48). **e**, Quantification of the fur status based on the evaluation score (0–3, where 0 indicates dense fur and 3 indicates thinner fur (UT *n* = 27; T *n* = 46; treatment lasted 4 months). **f**, Representative pictures of 24-month-old mice untreated or HK treated. **g**, Representative pictures of H&E staining of skin of 24-month-old animals. Insets show enlarged image of dashed box. **h**, Quantification of hair follicle diameter from sections of 24-month-old untreated or HK-treated mice (*n* = 6). **i**,**j**, Bone tissue percent (**i**) and bone mineral density (**j**) of the femurs of young (4 months old) and 24-month-old animals after 4 months of treatment (Bone tissue %, Young *n* = *6*; UT *n* = 8; T *n* = 8. Bone mineral density, Young *n* = *6*; UT *n* = 7; T *n* = 8). **k**, Quantification of proteoglycan loss (Young *n* = 8; UT *n* = 12; T *n* = 12). **l**, OARSI score quantification (Young *n* = 8; UT *n* = 10; T *n* = 12). **m**, Representative images of Safranin staining for the detection of proteoglycan-rich cartilage of young (4 months old), 24-month-old untreated and 24-month-old treated animals after 4 months of treatment (top images observed at ×10 magnification, bottom (area indicated in dashed box in upper image) at ×40). **n**, Results of grip strength assay performed on young (3-month) and old (24-month) animals after 4 months of treatment (Young *n* = 10, UT *n* = 5, T *n* = 9). **o**, Quantification of tibialis muscle fiber CSA (Young *n* = 7; UT *n* = 16, T *n* = 15). **p**, Representative pictures of tibialis muscle sections with dystrophin immunofluorescence from young (4 months old), 24-month-old untreated or 24-month-old HK-treated mice after 4 months of treatment. **q**, Representative pictures of H&E staining of kidney sections of young (4 months old), 24-month-old untreated and 24-month-old treated animals after 4 months of treatment. Insets show enlarged image of dashed box. **r**, Quantification of glomeruli diameter (Young *n* = 7; UT *n* = 6; T *n* = 6). **s**, Representative pictures of PSR staining of kidney sections of young (4 months old), 24-month-old untreated and 24-month-old treated animals after 4 months of treatment. Insets show enlarged image of dashed box. **t**, Quantification of percentage of fibrotic area in kidney sections (Young *n* = 6; UT *n* = 6; T *n* = 9). **u**, Kidney mRNA expression of *Fn1*, *Col1a1* and *Col3a1* (each dot represents a different animal; Young n = *8*, UT *n* = 10, T *n* = 14). Data presented in **d**, **h**, **i**, **j**, **k**, **l**, **n**, **o**, **r**, **t** and **u** are presented as mean ± s.e.m. Data presented in **e** are presented as a violin plot. Statistical test used in **b** and **c**: log-rank test. Statistical test used in **e**, **h** and **j**: two-tailed unpaired *t*-test. Statistical test used in **i**, **k**, **l**, **n**, **r**, **t** and **u**: one-way ANOVA with Holm–Šidák’s multiple comparisons test. Statistical test used in **o**: Kruskal–Wallis test with Dunn’s multiple comparisons test. *P < 0.05; **P < 0.01; ***P < 0.001; ****P < 0.0001; NS, nonsignificant. Exact *P* values found in source data files. Source data

**Extended Data Fig. 1:**
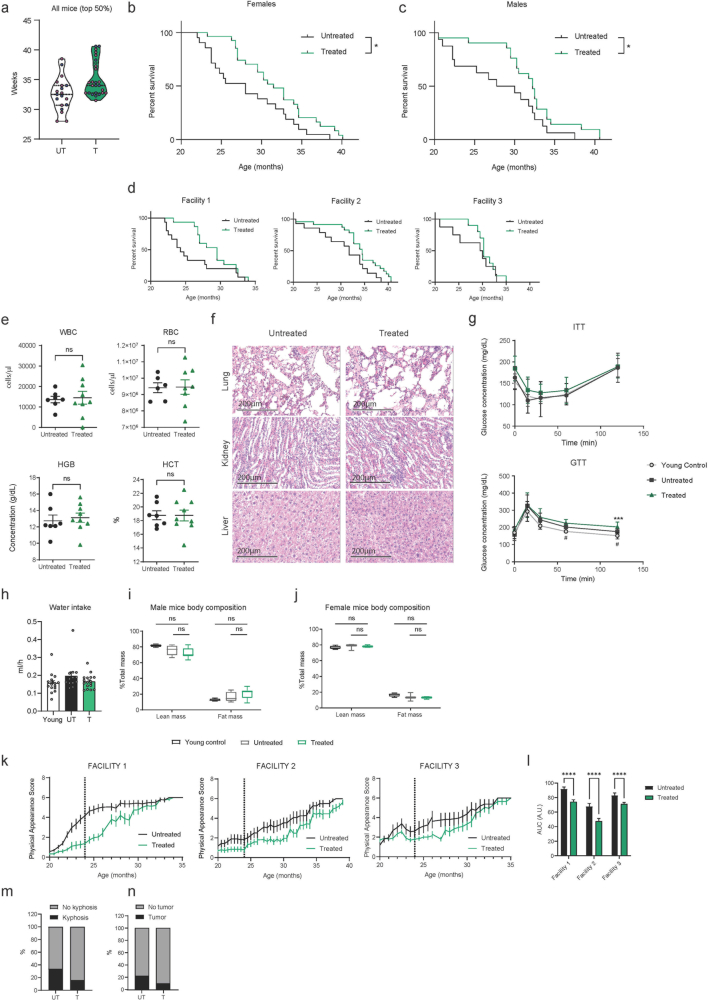
Haenkenium expands healthspan and lifespan in mice and does not show toxicity in vivo. **a**, Lifespan comparison of the most longest-lived mice from untreated and treated groups (top 50%); pink marks indicate females, blue marks indicate males (Female UT *n* = 11; Female T *n* = 14; Male UT *n* = 8; Male T *n* = 11). **b**, Survival curves for female mice (UT *n* = 21, T *n* = 27). **c**, Survival curves for male mice (UT *n* = 16, T *n* = 21) animals. **d**, Survival curves of animals hosted and treated in the different facilities (Facility 1 Treated *n* = 15; Untreated *n* = 15; Facility 2 Treated *n* = 23; Untreated *n* = 14; Facility 3 Treated *n* = 10; Untreated *n* = 8). **e**, Hematological parameters of treated and untreated animals: white blood cells (WBC), red blood cells (RBC), hemoglobin concentration (HGB), hematocrit (HCT) (WBC, HGB, HCT; UT *n* = 7, T *n* = 9. RBC, UT *n* = 6, T *n* = 8). **f**, Representative images of lung, kidney and liver of 24-month old treated and untreated animals after 4 months of treatment. No significant differences can be spotted among groups (photomicrographs representative of similar results from *n* = 4 mice per group). Scalebar: 200 μm. **g**, Glucose concentration after insulin injection (ITT upper panel) and after glucose injection (GTT, lower panel) (UT *n* = 7, T *n* = 9, Young *n* = 10). **h**, Average water intake during the whole day, ml/h (Young *n* = 15, UT *n* = 13, T *n* = 17). **i**-**j**, Lean and Fat mass were analyzed by EchoMRI in male (**i**) (UT *n* = 5. Treated *n* = 7. Young Control *n* = 4) and female (**j**) (UT *n* = 3, Treated *n* = 3, Young Control *n* = 4) animals. **k**, Physical appearance score progression of animals hosted and treated in the different facilities (Facility 1 Treated *n* = 15; Untreated *n* = 15; Facility 2 Treated *n* = 23; Untreated *n* = 14; Facility 3 Treated *n* = 10; Untreated *n* = 8). **l**, Area under the curve (AUC) analysis for data in **e**. (A.U. Arbitrary Units). **m**, Percentage of animals with kyphosis after 4 months of treatment (UT *n* = 27, T *n* = 46). **n**, Tumor prevalence after four months of treatment (UT *n* = 9, T *n* = 10). Data presented in panels **e**, **g**, **h**, **i**, **j**, **k**, **l** are presented as mean ± SEM. Statistical test used in **b** and **c**: Log-rank test. Statistical test used in **e**, **l**: Two-tailed unpaired t-test. Statistical test used in **g**: Two-way RM ANOVA with Dunnet’s multiple comparisons test. Statistical test used in **h**: One-Way ANOVA with Tukey’s test. Statistical test used in **i**, **j**: Two-way ANOVA with Dunnet’s multiple comparisons test. *=p < 0.05; ***=p < 0.001; # =p < 0.05 (UT vs Young Control). Exact p values found in source data files. Source data

To assess the effects of SH on longevity in vivo, we utilized a standardized plant extract referred to as HK^28^, which was administered in drinking water to aged mice starting at 20 months of age until the end of life (0.5 mg kg^−1^ body weight) (Fig. 1a). HK-treated animals had a median lifespan of 32.25 months, compared with 28 months in the untreated group (Fig. 1b,c). In both female and male mice, we observed a significant lifespan extension, thereby suggesting that the effect of HK treatment was sex-independent (Extended Data Fig. 1a–c). Mice were housed in three different animal facilities, thereby minimizing bias while increasing the reproducibility and generalizability of our findings (Fig. 1a, Extended Data Fig. 1d and Supplementary Table 1). In these experiments, both control and HK mice had ad libitum access to the same chow. Thus, the improved survival of HK mice was not a consequence of the low survival of control animals, differences in diet or housing conditions. HK administration was safe, and treatment did not result in alterations of hematopoietic parameters or organ toxicity, as assessed by histopathological evaluation of the skin, liver, kidney and lung of animals treated (Extended Data Fig. 1e,f). Moreover, HK treatment, compared with control, did not induce changes in glucose concentration after either insulin or glucose injection, changes in daily average water intake, or alterations in lean and fat mass in male and female mice (Extended Data Fig. 1g–j).

Aging is a complex systemic set of alterations that progressively accumulate over organismal lifespan detected at the phenotypic level^7^, including changes in physical appearance. To better characterize the effect of HK on the healthspan of treated mice, we developed a multiparametric score—the Physical Appearance Score—including the in vivo evaluation of fur status, kyphosis, eye cataracts and the presence of palpable tumors in the experimental mice. The evaluated parameters demonstrated that HK-treated animals showed a reduction in the severity of age-related phenotypes over time compared with the untreated group—an effect that was observed regardless of the animal facility (Fig. 1d and Extended Data Fig. 1k,l). The effect was particularly evident in kyphosis, tumor development and animal fur status (Fig. 1e,f and Extended Data Fig. 1m,n). Collectively, these results indicate that HK increases healthspan and lifespan in mice, with no observed toxicity.

### HK treatment improves phenotypes of aging in several tissues

HK treatment improved the aspect of fur in both male and female mice compared with controls (Fig. 1e,f). Fur and hair loss is associated with miniaturization of the hair follicle, which is a common phenotype during aging^29–31^. We then sought to understand whether HK administration was able to counteract this phenotype. Hematoxylin and eosin (H&E) staining of skin tissues revealed a significant amelioration in follicle miniaturization as measured by hair follicle diameter in the HK treated group (Fig. 1g,h).

**Extended Data Fig. 2:**
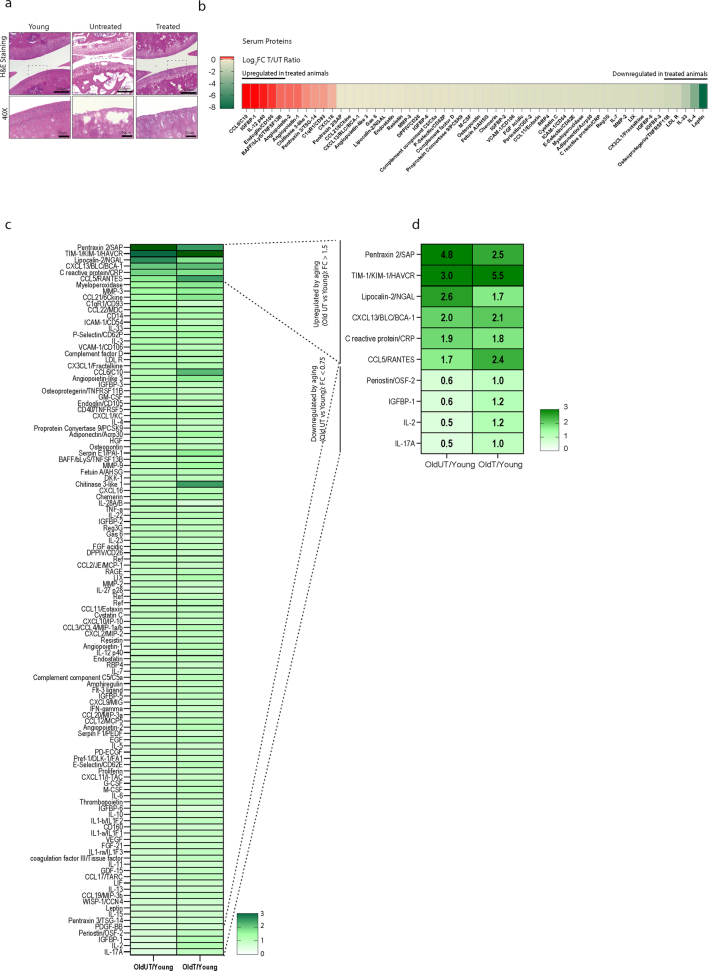
Haenkenium protects joints and modulates cytokine expression in tissues. **a**, Representative micrographs of Hematoxylin & Eosin staining of the knee joints of young (4-month-old), 24-month-old untreated, and 24-month-old treated animals after 4 months of treatment (top images observed at 10x magnification, bottom at 40x). Scalebar = 200 μm, inserts 50 μm. Photomicrographs representative of similar results from Young *n* = 8; UT *n* = 10; T *n* = 12 independent experiments. **b**, Proteome profiler analysis of cytokines expressed in serum of 24-month old untreated and animals, after 4 months of treatment. The heatmap represents the log_2_ fold change of the comparison between treated and untreated animals. **c**, Proteome profiler analysis of cytokines of kidney protein lysates from young (4-month-old), 24-month-old untreated, and 24-month-old treated mice after 4 months of treatment. The heatmap represents the fold change (FC) of the comparison of untreated/treated old mice relative to young. **d**, Highlight of proteins that are increased (FC > 1.5) and decreased (FC < 0.75) by aging, respectively. Source data

As musculoskeletal frailty is a crucial issue in aged subjects^32^, we investigated the effects of treatment on bone, joint and muscle health in animals. Bone structure analysis through microcomputed tomography and histology showed an increased bone mineral density (BMD) in the femurs of 24-month-old mice (Fig. 1i,j) and decreased age-driven degenerative alterations of the knee cartilage as determined by H&E and safranin staining (Fig. 1k–m and Extended Data Fig. 2a). We next assessed the forelimb grip strength of young and old mice untreated or treated with HK. Old animals showed a reduced grip strength compared with their younger counterparts, whereas treatment significantly ameliorated the loss of strength in old mice (Fig. 1n). In addition, we found that aged mice displayed significantly lower myofiber cross-sectional area (CSA) compared with young mice, with a trend toward improvement by HK treatment as determined by immunofluorescence of dystrophin and 4,6-diamidino-2-phenylindole (DAPI) of anterior tibialis muscle (Fig. 1o,p).

The aging process often results in a decline in renal function^33^. In this regard, H&E staining of paraffin-embedded kidneys from treated mice showed decreased glomeruli diameters to a level comparable with that of young mice (Fig. 1q,r). Kidney of untreated 24-month-old mice showed increased size of the Bowman’s capsule—an established alteration in renal aging^34,35^—as determined by measuring glomerular diameter. This effect was attenuated by HK treatment. Furthermore, HK-treated mice showed a marked decrease in kidney fibrosis compared with untreated mice, as detected by picro-sirius red (PSR) staining and real-time quantitative PCR with reverse transcription (RT-qPCR) analysis for extracellular matrix proteins, such as collagen (*Col1a1*, *Col3a1*) and fibronectin (*Fn1*)^36^ (Fig. 1s–u). HK treatment also decreased the levels of several circulating serum markers associated with renal disease, such as Cystatin C^37^ (Extended Data Fig. 2b). Proteome profiler analysis of kidney lysates from young, old untreated and old treated mice also revealed that treatment with HK reverted the age-related increase of Pentraxin-2/SAP and Lipocalin-2/NGAL—two proteins associated with inflammation and kidney damage, respectively^38,39^ (Extended Data Fig. 2c,d). Taken together, these data demonstrate that HK treatment in aged mice can alleviate several age-related phenotypes in vivo, such as hair loss, musculoskeletal frailty and renal fibrosis.

### HK effects on molecular pathways related to aging

**Fig. 2:**
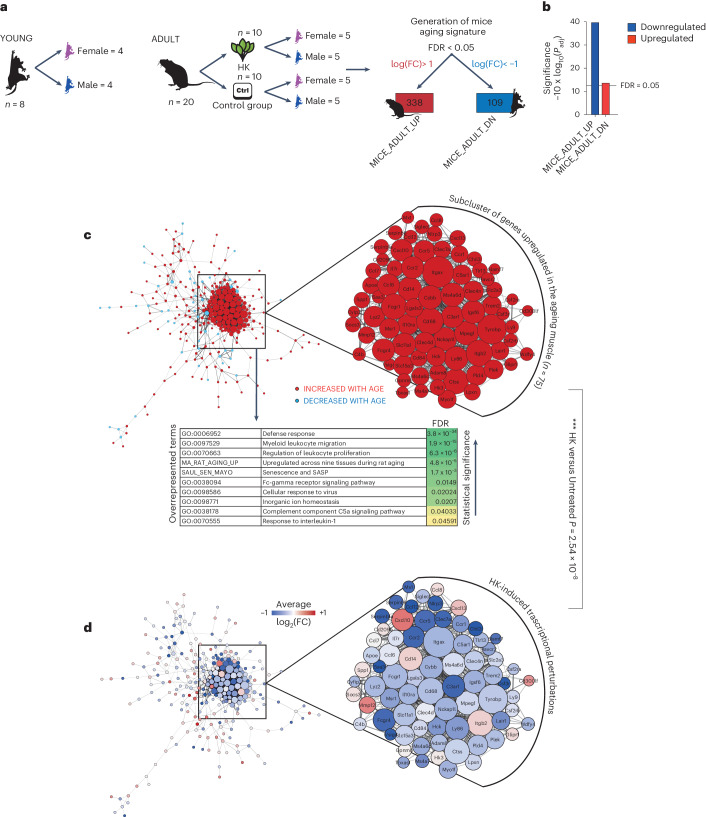
HK partially reverts age-related gene expression signature in mouse muscles. **a**, Experimental design of the bulk RNA-seq experiment. Total RNA was extracted from the gastrocnemius muscle of young (4 months old), old untreated (24 months old) and old treated (24 months old, 4 months of treatment) animals (Young *n* = 8, UT *n* = 10, T *n* = 10). **b**, Barplot representing GSEA results between old HK-treated animals and age-matched controls. Tested gene sets are those obtained by differential expression between older animals and younger controls (false discovery rate < 0.05, log_2_(FC) <−1 or >1). Barplots indicate a significant counteraction of HK towards age-induced perturbation in muscle tissue. **c**,**d**, Protein–protein interaction network including DEGs (false discovery rate < 0.05, log_2_(FC) <−1 or >1), as determined from muscle tissue of older mice compared with younger counterparts (**c**) or from the comparison between HK-treated animals versus aged-matched controls (**d**) showing close-up view of the genes most upregulated in aging. The table of overrepresentation analysis in **c** indicates gene sets significantly upregulated in the aging muscle.

To assess the mechanisms through which HK affects cellular functions to increase lifespan in vivo, we performed bulk RNA sequencing (RNA-seq) on the gastrocnemius muscle of treated mice (Fig. 2a). By using DESeq2, we first performed differential expression analysis between muscle-specific transcriptomes of old versus young animals to derive an aging signature. This aging signature included differentially expressed genes (DEG) that were either upregulated or downregulated by comparing old versus young mice. Next, we used this aging signature to study the transcriptional perturbations occurring in the muscle of HK-treated mice compared with age-matched adult untreated controls (24-month-old mice). First, we identified genes that are upregulated (338 DEGs) or downregulated (109 DEGs) during aging and found that HK-treated mice show downregulation of the genes upregulated in aged mice and vice versa (Fig. 2b). Among these genes, we identified a principal cluster of genes that is upregulated in old untreated mice compared with young mice (Fig. 2c). Notably, the cluster of genes upregulated in aging were overrepresented in pathway signatures related to inflammation, immune activation and senescence or SASP, including the recently described SAUL_SEN_MAYO gene set^40^. Subsequently, HK treatment strongly decreased the aging signature compared with untreated mice (Fig. 2d and Supplementary Table 2).

**Extended Data Fig. 3:**
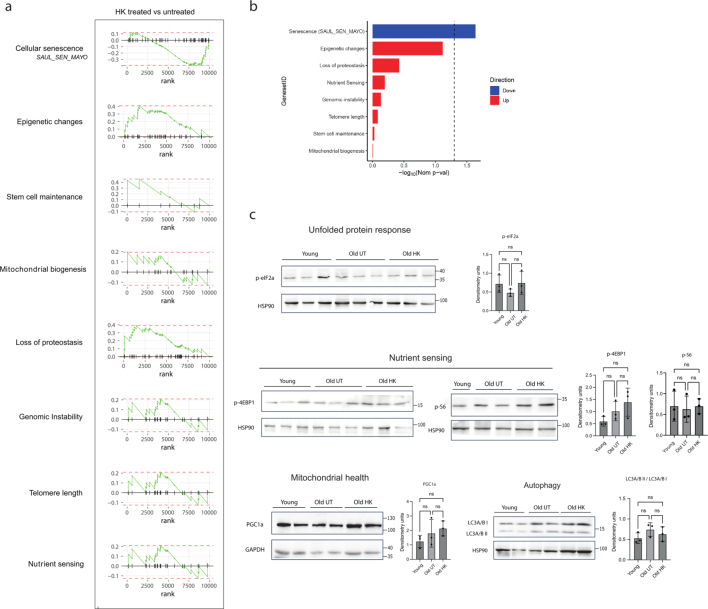
Haenkenium treatment does not affect several hallmarks of aging in muscle transcriptome and in kidneys. **a**, Gene Set Enrichment Analysis (GSEA) of manually curated gene sets corresponding to different aging-related pathways in muscle tissue from HK-treated and untreated 24-month-old animals after 4 months of treatment (*n* = 10). **b**, Results of GSEA analysis shown in (**a**). Barplots indicate the significance of the comparison between HK-treated and untreated 24-month-old animals after 4 months of treatment. **c**, Western blot and densitometric analysis of of p-eIF2$\alpha$, p-4EBP1, p-S6, LC3A/B and PGC1$\alpha$ from the kidney of young (4-month-old), 24-month-old untreated, and 24-month-old treated animals after 4 months of treatment. (*n* = 3). Data in **c** are presented as mean ± SD. Statistical test used in **c**: One Way ANOVA with Tukey’s test. ns = not significant. Exact p values found in source data files. Source data

Intrigued by these results, we performed additional analyses for other pathways involved in aging in the whole sequencing of the gastrocnemius muscle. In addition to the senescence gene set SAUL_SEN_MAYO, we evaluated additional aging-related pathways by performing gene set enrichment analysis (GSEA) on manually curated gene sets for nutrient sensing, loss of proteostasis, stem-cell maintenance, mitochondrial biogenesis, telomere length, genomic instability and epigenetic changes. Among these, we found that HK-treatment downregulated SAUL_SEN_MAYO (Extended Data Fig. 3a,b). On the contrary, nutrient sensing, loss of proteostasis, stem-cell maintenance, mitochondrial biogenesis, telomere length, genomic instability and epigenetic changes were not affected by HK treatment (Extended Data Fig. 3a,b and Supplementary Table 3).

Furthermore, we examined various processes via western blot analysis, including unfolded protein response (evaluated through p-eIF2α levels), nutrient sensing (assessed by p-4EBP1 and pS6 levels), autophagy (measured by LC3A/B I and LC3A/B II levels) and mitochondrial health (determined by PGC1α levels). In line with our transcriptomic assessments, none of these investigated processes exhibited perturbations due to aging or subsequent HK treatment (Extended Data Fig. 3c). In sum, our data show that HK treatment modulates a transcriptomic signature of aging in the gastrocnemius muscle associated with inflammation and senescence.

### Suppression of senescence by HK treatment

**Fig. 3:**
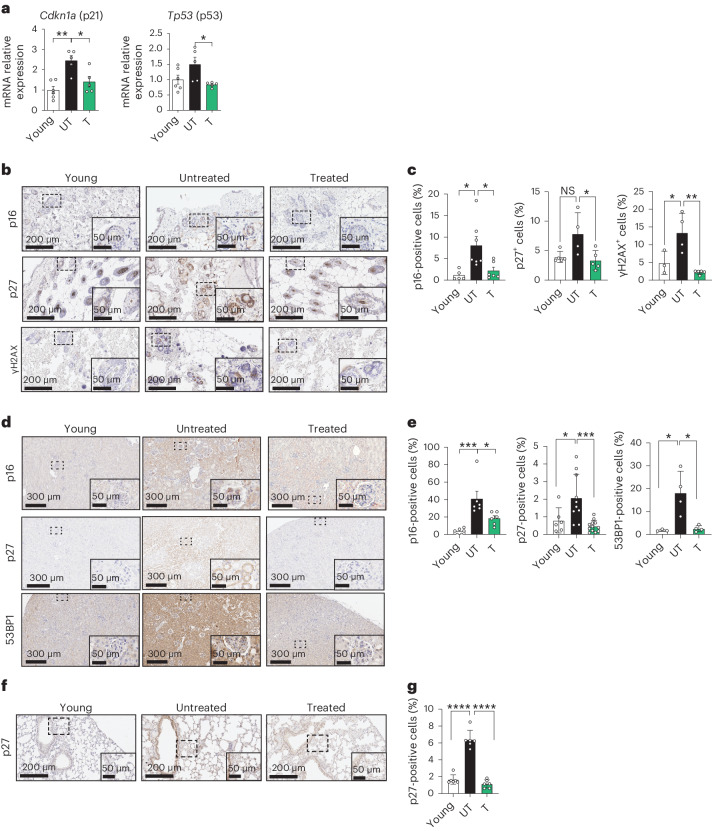
HK improves senescence in mice. **a**, mRNA levels of *Cdkn1a* and *Tp53* in the gastrocnemius muscle of young (4 months old), old untreated (24 months old) and old treated (24 months old, 4 months of treatment) animals (Young *n* = 6, UT *n* = 5, T *n* = 5). **b**, Representative images of p16, p27 and γH2AX IHC analysis performed on skin sections of young (4 months old), old untreated (24 months old) and old treated animals (24 months old, treatment for 4 months). Insets show enlarged image of dashed box. **c**, Quantification of p16, p27 and γH2AX-positive cells in skin sections from different animals (each dot represents a different animal; p16 Young *n* = 5, UT *n* = 7, T *n* = 6; p27 Young *n* = 5, UT *n* = 4, T *n* = 5; γH2AX Young *n* = 3, UT *n* = 4, T *n* = 5). **d**, Representative images of p16, p27 and 53BP1 IHC analysis performed on kidney sections of young (4 months old), old untreated (24 months old) and old treated (24 months old, 4 months of treatment) animals. Insets show enlarged image of dashed box. **e**, Quantification of p16-, p27- and 53BP1-positive cells in kidney sections from different animals (each dot represents a different animal; p16 Young *n* = 6, UT *n* = 6, T *n* = 6; p27 Young *n* = 6, UT *n* = 10, T *n* = 12; 53BP1 Young *n* = 3, UT *n* = 4, T *n* = 4). **f**, Representative pictures of p27 immunohistochemistry performed on lung sections of young (4 months old), old untreated (24 months old) and old treated (24 months old, 4 months of treatment) animals. Insets show enlarged image of dashed box. **g**, Quantification of p27-positive cells in lung sections from different animals (each dot represents a different animal; *n* = 6). Data in **a**, **c**, **e**, **g** are presented as mean ± s.e.m. Statistical test used in **a**, **c**, **e** and **g**: one-way ANOVA with Tukey’s multiple comparisons test. *P < 0.05; **P < 0.01; ***P < 0.001; ****P < 0.0001; NS, nonsignificant. Exact *P* values found in source data files. Source data

We corroborated our findings on downregulation of the senescence signature in the muscle by performing RT–qPCR on muscle samples, revealing that HK treatment reduced mRNA expression of *Cdkn1a* and *Tp53* (Fig. 3a). Given the downregulation of senescence-associated genes and gene sets in muscle by HK, we explored features of senescence in other organs described to be ameliorated from phenotypic aging by HK treatment. For this reason, we performed immunohistochemical (IHC) analysis on 24-month untreated and treated mice for different senescence markers such as p16, p27, γH2AX and 53BP1 (ref. ^41,42^). IHC staining performed on the skin for p16, p27 and γH2AX revealed these markers to be upregulated during aging, and HK treatment reverted this phenotype (Fig. 3b,c). We also investigated the expression of these markers in the kidney—another tissue affected by aging. The senescence markers p16, p27 and 53BP1 were upregulated markedly during aging while being attenuated by HK treatment (Fig. 3d,e). Moreover, as the gerosuppressant effect of SH was previously shown in lung fibroblasts in vitro^24^, we also investigated its efficacy in vivo by exploring, by IHC, p27 expression in the lungs of 24-month-old HK-treated mice. Similar to what was observed in the skin and kidneys, HK treatment attenuated age-associated increases in p27 levels (Fig. 3f,g). Together, these results demonstrate that HK treatment decreases the levels of several markers of senescence driven by aging in different tissues in vivo.

### HK protects against doxorubicin-induced senescence and cardiotoxicity

**Fig. 4:**
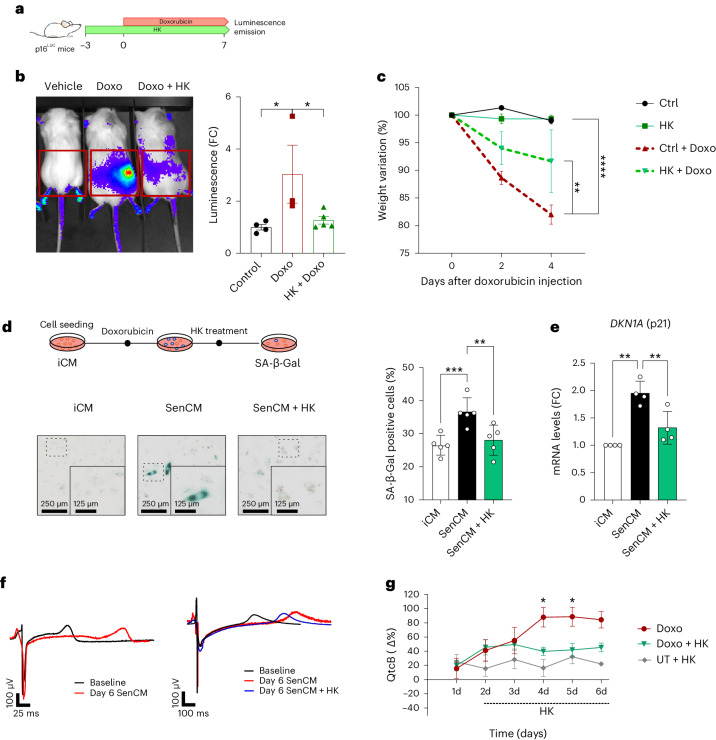
HK protects against Doxo-induced senescence and cardiotoxicity. **a**, Schematic representation of the experimental design. **b**, Left, representative images of luciferase detection in untreated and Doxo-injected animals (20 mg kg^−1^), with or without HK pretreatment (3 days, 0.5 mg kg^−1^). On the right, histograms show relative luminescence induction as FC (Control *n* = 4, Doxo *n* = 3, HK + Doxo *n* = 5). **c**, Animal weight loss measured 2 and 4 days after Doxo injection (*n* = 3). **d**, Representative images of SA-β-Gal staining of induced cardiomyocytes, with Doxo (SenCMs) or without (iCMs) or with 100 μg ml^−1^ of HK (*n* = 5) (left), and its quantification (right). Insets show enlarged image of dashed box. **e**, mRNA expression of *CDKN1A* (p21) as determined by RT-qPCR (*n* = 4). **f**, HK effect on SenCMs field potentials. Examples of QT interval measured in field potential recorded from different experimental conditions in comparison with baseline (black traces) at day 0 and day 6 are shown in subsets. **g**, The electrical activity of spontaneously beating iCMs was recorded using MEA for 6 consecutive days at baseline, after Doxo treatment (red line), following exposure of SenCM to HK (Doxo + HK; green line) and after exposure of iCM to HK (UT + HK; gray line) (*n* = 5 as means of different recordings). Data in **b**, **c**, **d**, **e**, **g** are presented as mean ± s.e.m. Statistical test used in **b**: one-way ANOVA with Holm–Šidák’s multiple comparisons test. Statistical test used in **c**: two-way ANOVA with Dunnettʼs multiple comparisons test. Statistical test performed in **d** and **e**: repeated measures one-way ANOVA with Tukey’s multiple comparisons test. Statistical test performed in **g**: two-tailed unpaired *t*-test analyses of Doxo versus Doxo + HK. *P < 0.05; **P < 0.01. Exact *P* values found in source data files. Source data

In addition to natural aging, several stressors, such as chemotherapy, can induce senescent cell accumulation in cancer and healthy tissues^43^. Systemic senescence accumulation is a principal side effect of chemotherapy and radiotherapy (TIS), which contributes to impaired quality of life of cancer patients^10^. Since HK can mitigate senescence in naturally aged mice, we also hypothesized that HK could prevent TIS. For this reason, we took advantage of doxorubicin (Doxo)—a potent chemotherapeutic agent used to treat various types of cancer, known to induce cellular senescence by causing DNA damage and oxidative stress^44^. We tested HK efficacy in the senescence-reporter mouse model p16^LUC^, in which the promoter of p16 controls expression of a luciferase reporter gene^45^. A single injection of Doxo was sufficient to induce senescence, as demonstrated by the increase in the luminescent signal detected 7 days after Doxo administration by intraperitoneal injection (Fig. 4a,b). However, HK treatment administered in the drinking water starting 3 days before Doxo administration (0.5 mg kg^−1^, oral gavage) was sufficient to successfully reduce the accumulation of p16-positive senescent cells in Doxo-treated mice and ameliorate body weight loss (Fig. 4b,c).

Doxo-induced senescence within cardiac tissues has been described previously as a key pathomechanism in Doxo-induced cardiac dysfunction^46^, and cardiac toxicity poses a constraint on the therapeutic use of Doxo^47^. As described previously^46^, treatment with a sublethal concentration (200 nM) of Doxo was used to induced senescence in human-induced cardiomyocyte cells (iCM). Following senescence induction, iCM were treated with HK, which significantly prevented iCM senescence as determined by SA-β-Gal staining (Fig. 4d) and reduced p21 mRNA to a level comparable with that of untreated cells (Fig. 4e).

Doxo drives cardiotoxicity by impairing the functionality of ion channels in iCM, leading to prolongation of the QT interval, which underlies arrhythmogenic cardiomyopathy in patients^46^. In Doxo-treated senescent iCM (SenCM), we found a significant prolongation of the QT interval corrected by Bazzett’s formula (QTcB) (Fig. 4f,g). The prolongation of QTcB in SenCMs was rescued almost entirely by HK treatment. Note that HK alone did not affect the electrophysiological properties of control iCM (Fig. 4g).

Taken together, we demonstrate that HK administration before chemotherapy treatment can attenuate the detrimental side effects of chemotherapy while preserving its therapeutic efficacy. HK treatment resulted in a marked decrease of systemic senescence accumulation, preventing cardiotoxicity driven by senescence and ultimately restoring normal cardiac function.

### Modulation of p16–CDK6 by the HK constituent luteolin in senescence

**Fig. 5:**
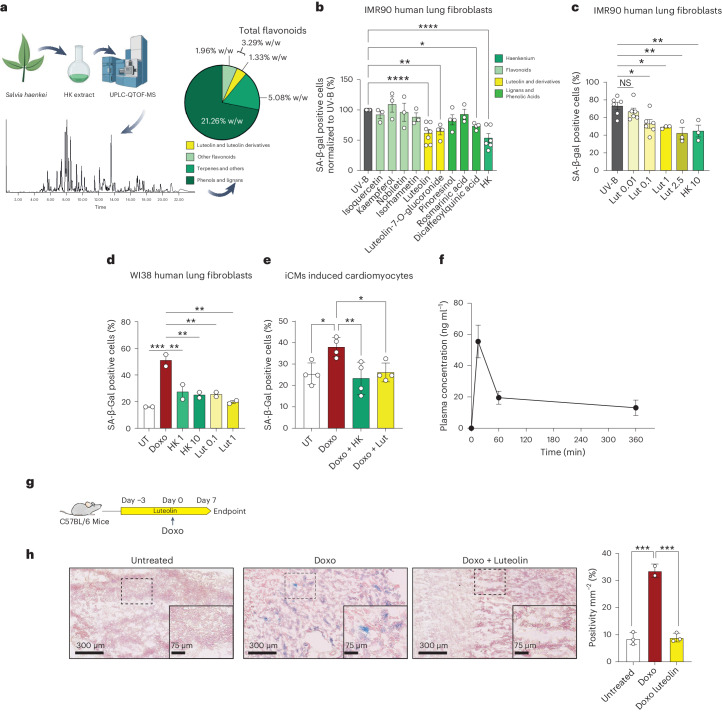
HK contains luteolin, which prevents stress-induced senescence. **a**, Top, Schematic representation of the experimental design; bottom, characterization of HK constituents by UPLC-QTOF-MS (Supplementary Table 4). **b**, Quantification of SA-β-Gal assay of UV-B-irradiated IMR90 fibroblasts, treated with the indicated compounds at 1 μM concentration or 10 μg ml^−1^ for HK (UV-B *n* = 9, isoquercetin *n* = 3, isorhamnetin *n* = 3, kaempferol *n* = 3, luteolin *n* = 7, luteolin-7-O-glucuronide *n* = 4, nobiletin *n* = 3, pinoresinol *n* = 4, rosmarinic acid *n* = 3, dicaffeoylquinic acid *n* = 4, HK *n* = 6 biological replicates). **c**, Quantification of SA-β-Gal-positive cells of UV-B irradiated IMR90 fibroblasts treated with different concentrations of Lut (0.01–2.5 μM) and HK (10 μg ml^−1^) (UV-B, Lut 0.01 μM, Lut 0.1 μM, *n* = 6 biological replicates; Lut 1 μM, Lut 2.5 μM, HK 10 μg ml^−1^, *n* = 3 biological replicates). **d**, Quantification of SA-β-Gal assay of Doxo-treated WI38 fibroblasts with or without Lut (0.1 and 1 µM) or HK (1 and 10 µg ml^−1^) (*n* = 2 biological replicates). **e**, Quantification of SA-β-Gal assay of Doxo-treated iCM cardiomyocytes treated with or without Lut (1 µM) or HK (100 µg ml^−1^) (*n* = 4 biological replicates). **f**, Luteolin plasma concentration in mice treated with a single dose of HK by oral gavage (0.5 mg kg^−1^) at 0, 15, 60 and 360 min (*n* = 3 animals per timepoint). **g**, Schematic representation of the experimental design. **h**, Representative pictures of SA-β-Gal staining (left) and quantification (right) on kidney sections. Insets show enlarged image of dashed box. (Untreated *n* = 3, Doxorubicin *n* = 2, Doxo + Luteolin *n* = 3 animals). Data in **b**, **c**, **d**, **e**, **f** and **h** are presented as mean ± s.e.m. Statistical test performed in **b**, **c**, **d**, **e**: one-way ANOVA with Dunnett’s multiple comparisons test. Statistical test performed in **h**: one-way ANOVA with Tukey’s multiple comparisons test. *P < 0.05; **P < 0.01, ***P < 0.001; ****P < 0.0001; NS, nonsignificant. Exact *P* values found in source data files. Image in Fig. 5a created in using BioRender.com. Source data

Since HK is a botanical extract containing several phytoconstituents, we performed ultra-high-performance liquid chromatography with quadrupole time-of-flight mass spectrometry (UPLC-QTOF-MS) characterization to identify the principal components. The three main categories of molecules in the extract were phenols/lignans, flavonoids and terpenes (Fig. 5a and Supplementary Table 4). We then explored a panel of nine constituents from each class of molecule.

We chose to focus on evaluating flavonoids—naturally occurring compounds found in many plant-based foods that have been shown to possess anti-aging properties by modulating cellular senescence and oxidative stress^15–17,48^. First, we tested a panel of the six most represented flavonoids (isoquercetin, kaempferol, nobiletin, isorhamnetin, luteolin and luteolin-7-O-glucuronide), one lignan (pinoresinol) and two phenolic acids (3,4-dicaffeoylquinic acid and rosmarinic acid) that are detectable in HK by evaluating their capability to affect senescence induction at 1 µM. Among the HK component-treated IMR90 fibroblasts, only luteolin (Lut), luteolin-7-O-glucuronide (LutG) and 3,4-dicaffeoylquinic acid (DQ) significantly attenuated UV-B radiation-induced SA-β-Gal staining to levels similar to HK-treatment (Fig. 5b). Notably, Lut is the most prevalent identified flavonoid aglycone and, together with its derivatives, account for 1.33% (w/w) of the extract (Supplementary Table 4). Thus, we decided to explore the effects of Lut and derivatives further.

**Extended Data Fig. 4:**
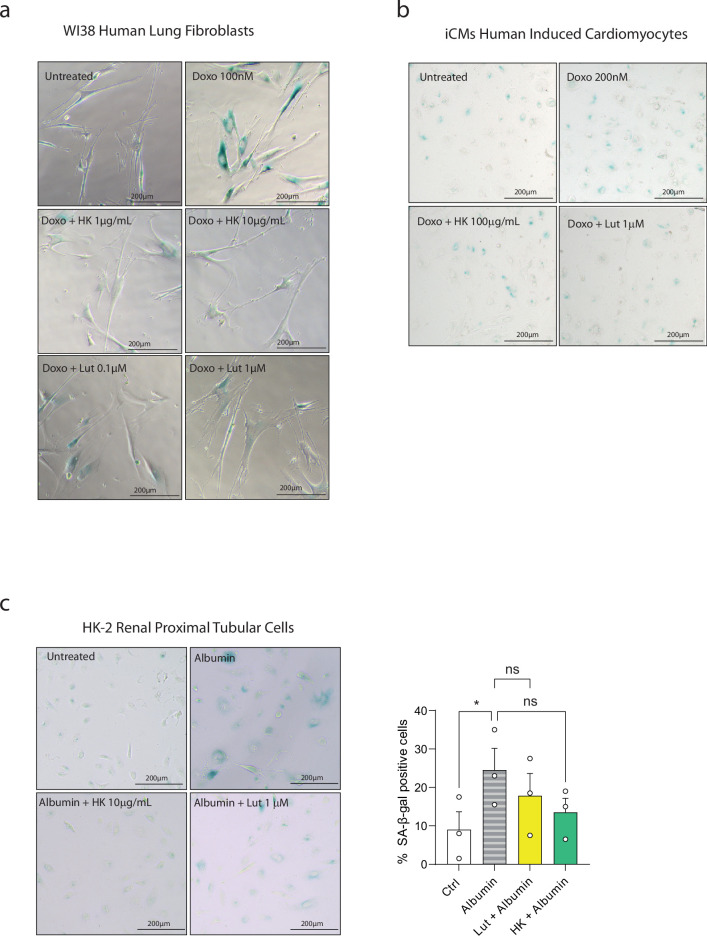
Anti-senescence effect of HK and luteolin in different cell types. **a-c**, Representative SA-β-Gal photomicrographs of WI38 fibroblasts (**a**, *n* = 2 biological replicates), iCMs (**b**, *n* = 4 biological replicates). **c**, Representative SA-β-Gal photomicrographs of HK-2 renal proximal tubular cells (right) and their quantification (*n* = 3 biological replicates). Scalebar = 200 μm. Statistical test used in **c**: One Way ANOVA with Holm-Šídák’s test. Quantifications for **a** and **b** are shown in Fig. 5. Source data

Lut treatment prevented SA-β-Gal-positive cell accumulation in a dose-dependent manner and to an extent similar to HK in UV-irradiated IMR90 fibroblasts (Fig. 5c). SA-β-Gal positivity was also prevented by Lut in Doxo-induced senescence to a similar extent as HK in WI38 fibroblasts and in Doxo-treated iCM (Fig. 5d,e and Extended Data Fig. 4a,b). In HK-2 renal proximal tubular cells induced to senescence by 100 mg ml^−1^ albumin treatment, there was a trend toward senescence amelioration by Lut and HK that was not significant (Extended Data Fig. 4c). Collectively, these data support our hypothesis that luteolin represents an active constituent within the HK phytocomplex by ameliorating SA-β-Gal positivity in different cell types induced by various external stressors.

The in vivo presence of Lut was confirmed with pharmacokinetic data. Mice were fed by oral gavage 0.5 mg kg^−1^ of HK, and plasma was taken at 15, 60 and 360 min post-treatment. Luteolin was detected in samples, with a C_max_ of approximately 55 ng ml^−1^ at 15 min post-treatment (Fig. 5f).

In an acute model of cellular senescence in vivo, C57BL/6 mice were pretreated with 0.5 mg kg^−1^ Lut 3 days before injection with 10 mg kg^−1^ Doxo. After 7 days, the animals were sacrificed and senescence was evaluated by measuring the percentage of SA-β-Gal-positive cells in the tissues (Fig. 5g). Treatment significantly protected against Doxo-induced SA-β-Gal positivity, suggesting an ameliorating effect of Lut against features of senescence at a relatively low dose (Fig. 5h).

**Extended Data Fig. 5:**
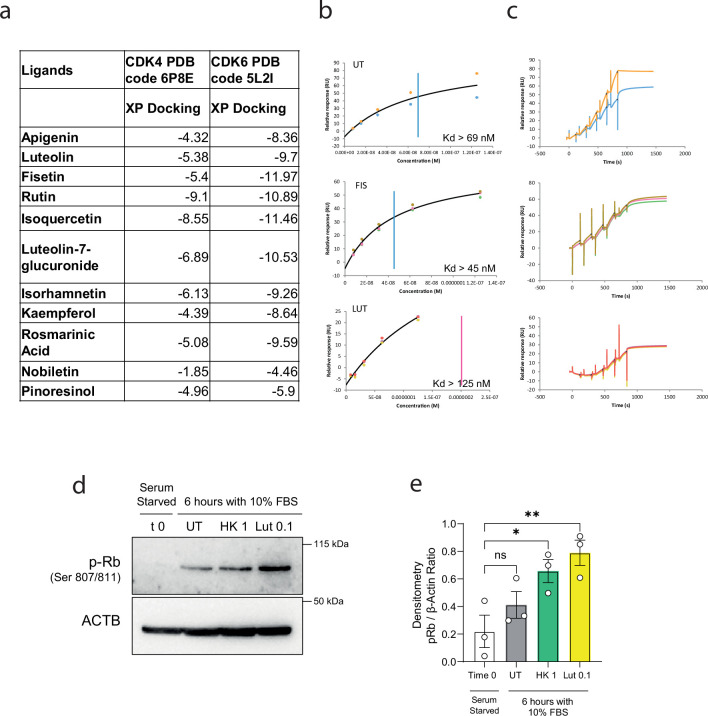
Chemogenomic analysis and Surface plasmon resonance. **a**, Evaluation of the GLIDE docking score for luteolin and other components of HK extract. **b-c**, Surface Plasmon Resonance Dose-response curve (**b**) and SPR sensograms (**c**) of the analysis of the SPR experiment to determine the CDK6-p16 Kd without any other molecules (UT) or in the presence of Luteolin (Lut) or Fisetin (Fis). Quantifications are shown in Fig. 6. **d**, Representative immunoblot of hyperphosphorylated Rb in untreated 48 hours serum starved IMR90 fibroblasts (t 0) or 6 hours following release from serum starvation induced cell cycle arrest with or without 1 μg/ml HK or 0.1 μM Lut (Lut 0.1). **e**, Densitometry of (**d**) (*n* = 3 biological replicates). Data are presented as mean ± SEM. Statistical test performed in **e**: One-way ANOVA with Dunnett’s test. *=p < 0.05; **=p < 0.01. Exact p values found in source data files. Source data

Given the capacity of Lut to inhibit cellular senescence, we wanted to characterize the molecular mechanisms underlying this effect. For this reason, we performed a docking analysis using publicly available datasets (ChEMBL; SIB; SEA Search Server). Among the proteins predicted to interact with luteolin on a similarity basis, we identified cyclin-dependent kinase 6 (CDK6). Through in silico analysis, we calculated the affinity between luteolin and CDK6 by evaluating the GLIDE Docking score^49^—an empirical scoring function approximating the ligand binding free energy. This analysis suggested that luteolin can bind CDK6 with a potent affinity, albeit with a similar predicted affinity as other flavonoids or HK constituents (for example, fisetin, apigenin) (Extended Data Fig. 5a).

**Fig. 6:**
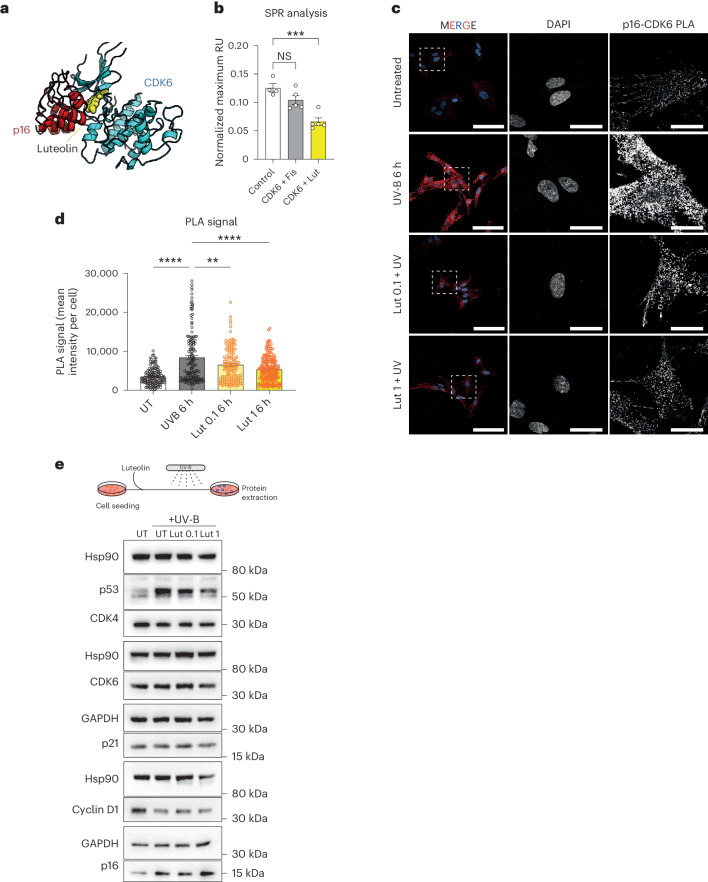
Disruption of the p16–CDK6 complex by luteolin. **a**, Atomistic model of the CDK6–p16 complex with luteolin obtained by the alignment of the results of docking calculations. **b**, SPR analysis to determine the CDK6–p16 interaction alone or with Fisetin (Fis) or Lut (Control *n* = 4, CDK6 + Fis *n* = 5, CDK6 + Lut *n* = 5 independent experiments). **c**, Representative pictures of PLA of CDK6–p16 interaction in UV-B-irradiated WI38 fibroblasts, pretreated with Lut (0.1 and 1 μM) for 1 h. Scalebars, 100 μm in merged panels, 25 μm in DAPI and p16–CDK6 PLA panels. Experiment performed twice independently with similar results. **d**, Quantification of PLA assay (UT *n* = 134, UV-B 6 h *n* = 142, Lut 0.1 6 h *n* = 122, Lut 1 6 h *n* = 170 cells observed over two independent experiments). **e**, Western blot analysis of the indicated proteins before and after UV-B irradiation of WI38 cells (*n* = 2 independent experiments). Data in **b** and **d** are presented as mean ± s.e.m. Statistical test performed in **b**: one-way ANOVA with Dunnett’s multiple comparisons test. Statistical test performed in **d**: one-way ANOVA with Bonferroni’s multiple comparisons test. **P < 0.01, ****P < 0.0001; NS, nonsignificant. Exact *P* values found in source data files. Source data

CDK6 regulates cell cycle progression from G_1_ to the S phase. Under damaging cellular conditions and in senescence, CDK6 activity is blocked by the interaction with p16 (ref. ^9^). Given that HK extract and its component luteolin delay the onset of senescence, we hypothesized that its binding with CDK6 might hinder the interaction between CDK6 and p16, making the kinase more refractory to p16-dependent inhibition. First, we generated an in silico three-dimensional model of the complex involving CDK6, p16 and luteolin. Luteolin was predicted to bind the interface of the two proteins, suggesting that the presence of luteolin with CDK6 might disrupt the interaction with p16 (Fig. 6a). We then set up a surface plasmon resonance (SPR) assay to study CDK6–p16 interaction: p16 was immobilized on the chip, and recombinant CDK6 was added at increasing concentrations. The assay was able to visualize the CDK6–p16 interaction (Fig. 6b and Extended Data Fig. 5b,c). On the contrary, the interaction was significantly disrupted in the presence of luteolin. Of note, another flavonoid with confirmed senotherapeutic properties and a similar chemical structure to luteolin, fisetin^13^, was not able to disrupt the interaction at the same concentration.

To validate disruption of the p16–CDK6 interaction as a mechanism of action for luteolin, we employed an in situ proximity ligation assay (PLA) in WI38 fibroblasts. Cells were pretreated for 1 h with different concentrations of luteolin (0.1 µM and 1 µM) before UV-B exposure. At 6 h after irradiation, cells were fixed and the p16–CDK6 interaction was confirmed by PLA and confocal microscopy (Fig. 6c). The mean fluorescence intensity (MFI) for each cell imaged was determined, enabling quantification of the interaction. A significantly higher MFI was observed for UV-B irradiated cells than for either untreated nonirradiated cells or with cells pretreated with luteolin before UV-B exposure (Fig. 6d). To confirm that the changes in MFI were not due to changes in protein expression, western blot analysis was done under the same conditions. UV-B exposure upregulated the expression of p16 across both untreated and luteolin-treated cells but did not affect the levels of CDK6 protein expression (Fig. 6e). As expected, other proteins involved in the cell cycle were downregulated by UV-B exposure, such as Cyclin D1 (CCND1). Taken together, these data show that luteolin can alter the interaction between p16 and CDK6 under senescence-inducing conditions, thereby potentially altering the development of the senescence phenotype.

Furthermore, we corroborated these findings by analysis of the immediate downstream target of CDK6 kinase activity: hyperphosphorylated Rb. IMR90 fibroblasts were serum-starved (0.2% fetal bovine serum (FBS)) for 48 h, resulting in quiescence and cell cycle synchronization^50^. After 48 h, cells were released from cell cycle arrest by the addition of a cell culture medium containing 10% FBS, with or without 1 μg ml^−1^ HK or 0.1 μM luteolin (Lut 0.1). After 6 h, cells were collected for western blot analysis of hyperphosphorylated Rb at residues phosphorylated by CDK6 (refs. ^51,52^) (Extended Data Fig. 5d,e). Compared with starvation-derived lysates (Time 0, t 0), HK- and Lut-treated cells displayed significantly higher phosphorylation of Rb than untreated cells, indicating that both treatments promoted the escape from cell cycle arrest in a CDK6-dependent manner. Taken together, these results suggest that luteolin and luteolin-containing botanical extracts such as HK can affect the CDK6–p16 axis, thereby altering the senescence phenotype.

## Discussion

The expanding aging population in most developed countries has led to rising societal and economic costs, creating a need to develop therapeutics that can promote healthy aging^5^. One promising avenue of research involves investigating the potential of botanical extracts, which are rich in phytoconstituents that can be studied or developed into healthspan-promoting agents^12^. Notably, naturally derived compounds that target cellular senescence have demonstrated the ability to improve healthspan and lifespan in various research models, spanning from cell cultures to invertebrates, rodents and humans^17^. Therefore, botanical extracts offer a promising area of study for developing therapeutics that can promote healthy aging and reduce the societal and economic burden of an aging population^3,11,12^. The removal of senescent cells has been shown to promote a healthy tissue microenvironment, as the accumulation of senescent cells results in the release of SASP, which contributes to low-grade inflammation that fuels age-related degenerative diseases^4^.

In the present study, we describe a natural extract that displays a senostatic/senomorphic effect. Treatment with HK—a standardized SH extract—ameliorated cell and tissue senescence. In vitro, HK treatment prevented UV-B and Doxo-induced senescent phenotypes, corroborating previous antisenescence effects of SH extract on cells^24,26^. We further extended these findings to mice, showing that daily treatment in drinking water with a relatively low dose of HK (0.5 mg kg^−1^) ameliorated age-related symptoms such as fur modifications, kyphosis, tissue accumulation of senescence and DNA damage markers, muscle strength and, notably, increased animal lifespan. Of great interest, we report that the working dosage is impressively low to exert gerosuppressant action in vivo; many studies examining natural products use higher doses of treatments, rendering their future development as medical foods, nutraceuticals or pharmaceuticals in humans impractical^53,54^. Furthermore, the treatment was started at a relatively late age (20 months), when mice already begin to display age-associated deterioration. Therefore, the promotion of healthspan by HK treatment is not a prerequisite for lifelong treatment, additionally underscoring its utility in development as a product for future human use.

With the aid of bulk RNA sequencing of gene expression in mouse gastrocnemius muscle, we identified a unique aging signature that included genes involved in inflammation, senescence and SASP. Importantly, the signature MICE_ADULT_UP which was upregulated in aged mice, was significantly downregulated by HK-treatment. This subcluster of genes upregulated in the aging muscle were significantly overrepresented in the gene set SAUL_SEN_MAYO, which was recently described to be strongly representative of aging and senolytic therapy^40^. Whereas SAUL_SEN_MAYO was downregulated in the HK versus untreated transcriptomic comparison, no other alterations in genes pertaining to established aging-associated pathways such as nutrient sensing, proteostasis, epigenetic regulation or stem-cell maintenance were significantly affected by treatment in the gastrocnemius muscle. Although further studies are required to rule out a possibility for HK-treatment to alter other mechanisms associated with healthspan extension, this evidence suggests a prominent role for the regulation of age-induced inflammation, SASP and senescence by treatment.

One of the strengths of our study is that we investigated the role of HK treatment against age-related senescence in several tissues as well as with different forms of senescence induction. We investigated TIS, as it has been widely reported that senescence accumulation within otherwise healthy tissues is an off-target effect of chemotherapy^43^. We used Doxo, which is a chemotherapeutic agent that induces senescence through heightened DNA damage and oxidative stress via inhibition of topoisomerase II (ref. ^47^). Doxo treatment in cancer patients results in deleterious side effects such as cardiac toxicity, fibrosis, inflammation, frailty and fatigue associated with senescence and/or SASP release^43,47^. Three days of pretreatment with a low dose of HK followed by a single injection of Doxo significantly reduced p16 expression in p16^LUC^ reporter mice. In a separate experiment, HK treatment ameliorated Doxo-induced senescence and cardiotoxicity in human iCM by relieving prolongated QT intervals—an underlying pathomechanism in arrhythmogenic cardiomyopathy in patients^46^. Additional research, however, should examine the role of HK in preventing chemotherapy-induced senescence caused by chronic exposure to Doxo to better mirror clinical use. Luteolin—a principal constituent of HK—has already been demonstrated to attenuate Doxo-induced cardiotoxicity and oxidative stress in vitro and in vivo^55,56^. Furthermore, we were able to corroborate this with luteolin-treated Doxo-induced senescent iCMs.

Previous research has demonstrated that HK exerted antioxidant effects and promoted RNA expression of SIRT1 (ref. ^26^). In the present study, we have explored in greater detail the mechanisms by which HK exerts antisenescence activity. The HK phytocomplex has been shown to contain numerous constituents, including polyphenols and terpenoids. Among these, we elected to further evaluate six flavonoids, two phenolic acids and one lignan for their antisenescence properties in UV-B-treated IMR90 human lung fibroblasts. Among the tested compounds, luteolin, luteolin-7-O-glucuronide and 3,4-dicaffeoylquinic acid treatment displayed the only significant efficacy in preventing senescence accumulation as measured by SA-β-Gal assay. From these, we pursued further study into luteolin. Luteolin displayed similar antisenescence properties to HK in fibroblasts and iCM. Notably, the amount of luteolin that we tested in vitro is within the relative concentration range that can be identified from the concentrations of HK that we assessed, demonstrating the relevance of this dataset. UPLC-QTOF-MS chemical analysis revealed that luteolin or its glycoside derivatives in the form of glucuronide, diglucuronide and rutinoside represent approximately 40% of total flavonoids in the HK extract. Considering that previous literature indicates that glycoside/glucuronide derivatives of luteolin are known to be hydrolyzed to their aglycone form in vivo^27,57^, the role of luteolin in the mechanism of action of HK can be considered important.

Luteolin is not unique to SH and is a common dietary compound found in many edible vegetables and herbs. Luteolin has a known diversity of biological properties and possesses antioxidant, anti-inflammatory and anticancer properties^27,57,58^ and has been shown to ameliorate senescence or SASP in stress- or treatment-induced models of senescence in different cell types through the modulation of sirtuins, the AMPK/mTOR pathway, and NF-κB^21,22,48^. As with natural products with pharmacological diversity, it is difficult to ascribe a single phenotypic effect (for example, senescence induction) to a singular pathway. Broadly, flavonoids and polyphenol-rich plant-based foods or extracts have been reported to have a wide range of longevity-promoting effects via several mechanisms of action beyond senescence regulation, including modulation of the gut microbiome to sparing NAD^+^ levels^59,60^. Admittedly, our present work focuses extensively on senescence modulation, where it is feasible that HK and luteolin may also promote other life- and health-extending mechanisms, in line with previous in vitro investigations demonstrating that SH can attenuate excessive ROS and upregulate SIRT1 mRNA^24,26^. Nonetheless, given the multitude of reported cellular activities of luteolin^27,57,58^, we propose a new mechanism by which luteolin could specifically alter the senescence phenotype.

Through computational molecular docking, luteolin was predicted to bind the cell cycle regulator CDK6 with good affinity. Furthermore, luteolin was modeled to disrupt binding of the cell cycle inhibitor p16 to CDK6, which we confirmed by SPR. Here, luteolin significantly reduced the capacity of p16 to bind CDK6. We also tested fisetin, a flavonoid with a similar chemical identity and notable senolytic effects^13^. Interestingly, although we and others^61^ demonstrated that fisetin binds to CDK6 with good affinity, fisetin did not impact the p16–CDK6 interaction as determined by SPR. One possibility is that fisetin could disrupt the p16–CDK6 complex as analyzed by SPR at a higher concentration; however, we were unable to test this due to solubility of high concentrations of the flavonoid in the system. Furthermore, we hypothesize that, while both luteolin and fisetin bind to the same pocket in CDK6, luteolin has two –OH moieties oriented toward the p16 binding interface, whereas fisetin has an –OH group facing an internal position. In theory, this could explain their different ability to disrupt the p16–CDK6 interaction, despite their shared affinity for CDK6. However, additional biochemical analyses will be required to best understand this mechanism of action.

To confirm our finding, we utilized an in situ PLA to assess the interaction between p16 and CDK6. The PLA is an immunoassay that detects protein–protein interactions with a resolution of 40 nm. WI38 fibroblasts exposed to UV-B irradiation displayed significantly heightened PLA signal, in line with heightened p16 protein expression consequent with UV exposure. However, in luteolin-pretreated cells, the signal was significantly attenuated, without changing protein expression of p16 or CDK6 in UV-B exposed cells. Hence, the observed changes in the PLA signal due to luteolin are probably resultant from disruption of the p16–CDK6 interaction, thereby confirming our computational and SPR data.

Indeed, we further assessed the potential for disruption of the p16–CDK6 complex by luteolin by studying the phosphorylation of the immediate downstream target of CDK6 signaling, Rb. After 48 h of serum starvation, cells were released from cell cycle arrest by adding a serum-containing medium with or without HK or luteolin. After 6 h, approximately midway through the G_1_ phase of the cell cycle, HK or luteolin significantly increased Rb phosphorylation at residues known to be phosphorylated by CDK6 (refs. ^51,52^).

As heightened p16 expression is both a marker of cellular senescence and a mechanism underlying the development of senescence through cell cycle inhibition, disruption of the p16–CDK6 interaction and subsequent phosphorylation of Rb may represent a mechanism by which natural products can prevent or delay the onset of senescence. Further studies should be conducted to confirm the importance of inhibiting the p16–CDK6 interaction in aging. Previous studies demonstrate knockdown of p16 prevents replicative senescence in normal human diploid fibroblasts^62^. Similarly, transgenic *INK-ATTAC* mice, where the elimination of p16-positive senescent cells can be induced, display ameliorated age-related tissue dysfunction and life extension, thus suggesting that targeting the p16–CDK6 axis can improve quality of life in age-related illness and disease^63^. Indeed, the selective elimination of p16-positive cells in *INK-ATTAC* mice resulted in improved skeletal muscle function and larger muscle fiber diameters in aging, which mirrors our own findings of preserved grip strength, thicker muscle fibers and reduced senescence markers in old HK-treated mice compared with untreated mice. Ultimately, the notion that treatment with a natural product may inhibit p16 is an attractive alternative approach in developing substances intended for human use. Given the role of p16 as a cell cycle inhibitor, a potential concern may be that its inhibition could contribute to a potential oncogenic effect. However, luteolin intake is not linked to cancer; on the contrary, it has demonstrated anticancer effects and, in our study among mice treated with HK, no mice displayed heightened tumor development compared with age-matched untreated controls^57^. This observation could furthermore be linked to the prevention of senescence accumulation, as senescent nontransformed fibroblasts promote malignant transformation of epithelial cells in a SASP-dependent manner^64^.

This study may have some limitations. First, although our work evaluated various parameters associated with aging and identified modulation of cellular senescence as a mechanism by which HK supports lifespan, it is important to note that organismal aging is molecularly and cellularly heterogeneous and it is feasible that other hallmarks of aging are regulated by HK-treatment in vivo. We previously identified a role for HK in modulating oxidative stress, and SIRT1 mRNA in vitro^24,26^, which should be investigated in further animal studies with HK.

Secondly, while luteolin was identified from HK as possessing antisenescence properties, HK is a chemically complex botanical preparation. Indeed, inherent to the study of complex botanical extracts is the presence of hundreds of confounding variables regarding the identification of an active constituent. Although we applied in vitro techniques to identify luteolin from a pool of HK-derived constituents as a molecule of interest with antisenescence activity, we did not exhaustively evaluate the potential for synergistic combinations of these molecules as they would exist within the botanical extract. Furthermore, although we have linked treatment of HK with improved lifespan and healthspan, it is only speculative to suggest that one constituent (for example, luteolin) is responsible for this effect, in the absence of in vivo studies with a single purified agent. Hence, further studies should investigate luteolin and luteolin derivatives in lifespan and healthspan extension.

Third, our work utilized a single inbred strain of mice, C57BL/6. Although this is common practice, the use of genetically heterogeneous mice could produce results that minimize the effects of strain-specific tendencies as research artifacts^65–67^.

Taken together, our research demonstrates that HK, a flavonoid-rich standardized natural plant extract from SH, can significantly ameliorate age-associated tissue dysfunction in association with a modulation of the senescence phenotype. We identify that HK contains luteolin and that luteolin may be responsible for some of the antisenescence effects of HK in association with the disruption of the p16–CDK6 interaction. We show that HK treatment exerts significant gerosuppressant and life-extending effects with a relatively low dose and that it displays a favorable safety profile. These data provide a good platform for future research and development of HK as a medical food or pharmaceutical for the treatment of age-related diseases.

## Methods

### Mice

Experiments were performed in the animal facilities of the Pharmacy and Pharmacology Department of the University of Padova and the Veneto Institute of Molecular Medicine (VIMM) under Italian national and EU directives (2010/63/EU) for animal research with protocols approved by Institute Ethical Committee and the Italian Ministry of Health (672/2019-PR and 336/2020-PR). Aging experiments were performed using C57BL/6 mice purchased from Charles River at the age of 78 weeks or with animals bred and aged in the animal facility of the Pharmacy Department (University of Padova). Mice were maintained at room temperature (20–22 °C), with humidity at 55 ± 10 %, and exposed to a 12-h daylight cycle. The mice were specified pathogen-free according to the FELASA list (FELASA 2014) with colonies monitored by a sentinel program.

p16^LUC^ mice (B6.Cg-Cdkn2atm3.1Nesh Tyr c-2J/Nci) from N. Sharpless^45^ were obtained from the NCI Mouse Repository. p16^LUC^ mice were maintained under specific pathogen-free conditions in the IRB animal facility of the Università della Svizzera Italiana (Bellinzona, Switzerland), and experiments were performed according to state guidelines and approved by the local ethics committee (Dipartimento della Sanità e Socialità, Esperimenti su animali, Canton Ticino), authorization numbers 30275 and 34293. Mice were housed in groups of a maximum of five animals per cage and fed standard laboratory chow and sterile water. Mice were housed in single-vented cages, maintained at room temperature (20–22 °C), with humidity at 55 ± 10 %, and exposed to a 12-h daylight cycle. We bred homozygous mice to generate animals with two copies of the transgene (p16^Luc/Luc^). Experiments were conducted on 10-week-old mice.

### Statistics and reproducibility

Data analyses were carried out using GraphPad Prism v.10.2.0 or Microsoft Excel 2018. The data are represented as mean ± s.d. or s.e.m. where indicated. The survival probability of Fig. 1b was calculated using the log-rank test. The Cox proportional hazards regression analysis in Supplementary Table 1 was performed with SAS v.9.4 (SAS Institute Inc.). Two-sided, paired or unpaired *t*-tests, according to the experimental setting, were used to compare two groups in the indicated experiments. In most cases, one-way analysis of variance (ANOVA) with Dunnett’s, Holm–Šidák’s or Tukey’s multiple comparisons tests or Kruskal–Wallis with Dunn’s post hoc test were used to compare three or more groups, and are specifically indicated in the figure legends. In most experiments, data were assumed to be normally distributed. When needed, the Shapiro–Wilk normality test was calculated using GraphPad. Statistical analyses performed on RNA-seq datasets are specifically described in their respective methods section. No statistical method was used to predetermine sample size, and group sizes were determined based on the results of preliminary experiments. All samples meeting proper experimental conditions were included in the analysis; samples were excluded for reasons of compromised specimen integrity/quality that would have negatively affected the analysis. Operators were not blind to the mouse treatment conditions due to the recognizable nature of the treatment administered in the drinking water. Analysis performed by pathologists including kidney H&E and kidney p16 IHC were performed blind. In all the other cases, data collection and analysis were not performed blind to the conditions of the experiments.

### HK plant material

IBSA Farmaceutici Italia S.r.l. provided the HK extract and its preparation was described previously by Scrima et al.^28^ Briefly, HK is an hydroethanolic extract of the aerial parts of SH with a standardized plant:dry extract ratio of 7:1. HK extract is available upon request to the corresponding author.

### In vivo administration of plant extract

HK extract was administered at the dosage of 0.5 mg kg^−1^ day^−1^ in drinking water. Mice treatment started at 20 months of age. HK extract was replaced and prepared fresh on a weekly basis.

### In vivo aging parameter studies

The physical appearance parameters were evaluated on male and female animals housed in three different animal houses. Mice were checked on a weekly basis for the evaluation of typical aging signs, in particular: the status of the fur (color, vibrance and presence of alopecia), whisker loss, development of kyphosis, cataracts and palpable tumors. Operators attributed a score to each parameter (0–3 for fur status; 0–1 for the presence/absence of other signs) to evaluate animal status. Mouse weight was monitored, and the animals were sacrificed when a reduction greater than 20% was assessed (humane endpoint). Body composition analysis in mice was performed via quantitative nuclear magnetic relaxometry, EchoMRI-100 (EchoMRI LLC), without anesthesia.

In some experiments, age-matched animals were sacrificed after 4 months of treatment. Mice euthanized after 4 months are not included in the survival. Due to the involvement of different facilities, different cohorts were scored by different operators. Operators received the same training, every cohort was evaluated by the same operators for the whole duration of the experiment; however, the scoring method was not cross-validated for subjective scoring, and operators could not be blind to treatment, as the extract was administered in the drinking water.

### Grip strength assay

The forelimb strength of mice was measured using the grip strength assay (BIOSEB, BIO-GS3) following manufacturer instructions.

### Immunohistochemistry

For immunohistochemistry (IHC), tissues were fixed in 10% formalin (Thermo Scientific, cat. no. 5701) and embedded in paraffin according to standard procedures. Tissue sections (4 µm) were exposed to two washes with OTTIX plus solution (Diapath, cat. no. X0076) and subsequent hydration with OTTIX shaper solution (Diapath, cat. no. X0096) followed by deionized water. Antigen unmasking was performed by heating sections in the respective pH solutions based on the antibodies used at 98 °C for 20 min. Subsequently, the sections were blocked for peroxidases and nonspecific binding of antibodies using 3% H_2_O_2_ (VWR chemicals, cat. no. 23615.248) and Protein-Block solution (DAKO Agilent technologies, cat. no. X0909) respectively for 10 min and then incubated for 1 h at room temperature with anti-p16 (rabbit, 1:1,000, Abcam, cat. no. ab211542), anti-p27 (rabbit, 1:1,000, Abcam, cat. no. ab32034), anti-γH2AX (rabbit, 1:1,000, Cell Signaling, cat. no. 9718) or anti-53BP1 (rabbit, 1:1,000, Abcam, cat. no. ab21083) diluted in antibody diluent (Invitrogen, cat. no. 003118). Following washing, sections were incubated in biotinylated goat anti-rabbit IgG antibody (Vector Laboratories, cat. no. BP-9100) for 30 min, then stained with Vectastain ABC Kit (Vector Laboratories, cat. no. PK-6100) and ImmPACT DAB horseradish peroxidase (HRP) Substrate (Vector Laboratories, cat. no. SK-4105). H&E staining was additionally performed on tissues according to standard protocol^68^. The diameter of either glomeruli^34^ in H&E-stained kidney sections or hair follicles in H&E-stained skin was quantified by using IAperio ImageScope—Pathology Slide Viewing Software (v.12.3.3).

### Myofiber CSA evaluation

Gastrocnemius muscles were immediately frozen and conserved in liquid nitrogen for gene expression and proteome analysis. Tibialis anterior muscles were frozen in isopentane, cooled in liquid nitrogen, and stored at −80 °C for morphological and histological studies. Serial cryosections (10 μm) from anterior tibialis muscles were mounted on lysine-coated glass slides (Superfrost), air-dried and stained either with H&E or immune-stained for dystrophin and DAPI for myofiber CSA evaluation by analyzing images captured an inverted microscope (DM6 B, Leica) with SMASH Stand Alone (MATLAB application). For immunofluorescence staining, muscle cryosections were first allowed to reach room temperature, fixed in 4% PFA, washed three times with PBS for 5 min and then permeabilized in 0.1% Triton X-100 in PBS for 2 min. Slides were then blocked with 10% goat serum in 0.5% BSA in PBS for 30 min. Muscle sections were then incubated with anti-dystrophin primary antibody (1:100; ThermoFisher Scientific, cat. no. PA5-32388) diluted in 2% goat serum in 0.5% BSA in PBS at +4 °C overnight. Sections were washed three times in PBS before being incubated for 1 h at room temperature with goat anti-rabbit CY3 secondary antibody (1:150 dilution, ThermoFisher Scientific, cat. no. A10520) diluted in 2% goat serum in 0.5% BSA in PBS. After washing, sections were stained with DAPI and mounted in Fluorescence Mounting Medium (Dako, cat. no. S3023).

### PSR staining

For PSR staining, sections were deparaffinized, rehydrated and incubated for 1 h in PSR staining solution. After washing two times in acidified water, sections were dehydrated and mounted.

### RT–qPCR

Total RNA was extracted from samples using TRIzol reagent from Invitrogen. cDNA synthesis was performed using High-Capacity cDNA Reverse Transcription Kit (Applied Biosystems, ThermoFisher Scientific). qPCR reactions were performed with Power SYBR Green PCR Master Mix (Applied Biosystems, ThermoFisher Scientific), and experiments were performed according to the manufacturer’s instructions on a QuantStudio 5 real-time PCR System (Applied Biosystems, ThermoFisher Scientific) and QuantStudio Design and Analysis Software v.1.4.3. For experiments with iCMs and SenCMs, TRI-Reagent (Sigma-Aldrich) was used for extraction, GoScript Reverse Transcription System kit (Promega) for reverse transcription and a CFX Connect Real-Time PCR Detection System (Bio-Rad) was used per manufacturer instructions. The threshold cycle (Ct) of each gene was defined automatically and normalized to the geometric mean control housekeeping genes GAPDH and RPL27 (ΔCt value). The Livak method was used to calculate the ΔΔCt^69^. Specific primers used are indicated in Supplementary Table 5.

### RNA-seq data processing of murine samples and batch effect correction

The overall quality of sequencing reads was evaluated using FastQC^70^. Sequence alignments to the reference mouse genome (GRCm38.p6) were performed using STAR (v.2.6.1c) in two-pass mode. Gene expression was quantified at the gene level by using the comprehensive annotations made available by Gencode (M25 GTF-File). Samples were adjusted for library size and normalized with the variance stabilizing transformation (vst) in the R statistical environment using DESeq2 (v.1.28.1) pipeline. Samples originated from two separated sequencing experiments, and unsupervised PCA analysis of the top 2,000 most variable genes showed a clear experiment-related batch effect along the first principal component. This bias was corrected using the removeBatchEffect function (limma package), whereas sex and age group were set as batch covariates. Subsequently, we adjusted expression data also for sex to identify gene expression changes occurring in a gender-independent manner.

### Generation of aging-related gene signatures

We performed differential expression analysis between muscle-specific transcriptomes of young mice and older animals using DESeq2, utilizing the Wald test for determining two-tailed *P* values. When comparing groups, we applied the embedded IndependentFiltering procedure to exclude genes that were not expressed at appreciable levels in most of the samples considered. We selected features showing an adjusted *P* value (*P*_adj_) < 0.05 and a log_2_ fold change (FC) >1 or <−1, which we used to build two signatures, namely MOUSE_ADULT_UP and MOUSE_ADULT_DN, consisting of 338 and 109 genes, respectively.

### Reversal of aging-related features in the muscle of HK-treated mice

We observed changes in gene expression in mice treated with HK when compared with age-matched adult controls. By sorting genes based on their *P* values, we conducted a GSEA utilizing the limma package (camera algorithm, nonparametric, rank-based). Our analysis revealed significant changes with custom-derived gene sets (MOUSE_ADULT_UP and MOUSE_ADULT_DN), indicating alterations contrary to those seen in normal aging due to HK treatment. For additional manually curated gene sets (Supplementary Table 3), we assessed statistical significance using Camera, with a nonparametric, rank-based approach and a presence filter requiring a baseMean ≥50. Normalized enrichment scores were calculated using GSEA preranked.

### Proteome profiler analysis

Cytokine analysis of mouse serum and tissue samples was performed using Proteome Profiler Mouse XL Cytokine Arrays (R&D Systems, cat. no. ARY028), following the manufacturer instructions. Analysis was performed using protein array analyzer (ImageJ).

### Histological evaluation of articulations

The methodology is described in Supplementary Methods.

### Microcomputed tomography analysis

The methodology is described in Supplementary Methods.

### Luminescence detection in Doxo-treated p16^LUC^ mice

p16^LUC^ reporter mice were monitored using IVIS imaging system (IVIS spectrum, Perkin Elmer) 7 days following Doxo injection. Xenolight D-luciferin (Perkin Elmer, cat. no. 122799) at 150 mg kg^−1^ was injected in mice 12 min before IVIS spectrum imaging.

### Cell lines

WI38 human lung fibroblasts and IMR90 human lung fibroblasts were obtained from ATCC (cat. nos. CCL-7 and CCL-186, respectively) and cultured in Minimum Essential Medium alpha (Gibco, cat. no. 32561029) supplemented with 10% FBS (Gibco, cat. no. 16000044) and 100 U ml^−1^ penicillin and 0.1 mg ml^−1^ streptomycin (Gibco, cat. no. 15140122). Normal human renal proximal tubular cells (HK-2) were purchased from ATCC (cat. no. CRL-2190), and cultured in DMEM containing 5 μg ml^−1^ insulin, 10 μg ml^−1^ human apotransferrin, 500 ng ml^−1^ hydrocortisone, 10 ng ml^−1^ epithelial growth factor, 6.5 ng ml^−1^ triiodothyronin, 5 ng ml^−1^ sodium selenite, 1% fetal calf serum, 25 IU ml^−1^ penicillin, 25 μg ml^−1^ streptomycin and 10 mM HEPES buffer.

### Human cardiomyocytes from induced-pluripotent-stem-cells

Human cardiomyocytes were derived from induced-pluripotent-stem-cell (iPS) using a cell line previously generated and established in our laboratory^46,71^. Differentiation of pluripotent cells into iCMs was obtained via WNT signaling pathway modulation. Briefly, at day 0, the iPS colony was plated in a 12-well Matrigel-coated plate in the presence of RPMI 1640 supplemented with B-27 minus insulin (ThermoFisher Scientific) and exposed to 4 μM CHIR99021 (Merck Millipore) for 48 h and 5 μM IWP4 (Merck Millipore) for a subsequent 48 h. On day 7, the medium was exchanged with RPMI 1640 with B-27 plus insulin (ThermoFisher Scientific). From days 10–17, iCMs were enriched by metabolic selection using a medium composed of RPMI 1640 without glucose (ThermoFisher Scientific), including 0.5 mg ml^−1^ human recombinant albumin, 0.2 mg ml^−1^ l-ascorbic acid 2-phosphate, and 4 mM lactate (Sigma-Aldrich). Afterward, iCMs were cultured in a maintenance medium at least to day 30 for further maturation.

### In vitro treatments

HK was dissolved in dimethylsulfoxide (DMSO, Sigma-Aldrich) and adjusted to the desired concentration in the cell culture medium. Individual constituents used in Fig. 5b were dissolved in DMSO and adjusted to a final concentration of 1 µM in MEMα for treatment. Tested compounds include 3,4-dicaffeoylquinic acid (MedChem Express, cat. no. HY-N0057), kaempferol (MedChem Express, cat. no. HY-14590), isorhamnetin (MedChem Express, cat. no. HY-N0776), isoquercetin (MedChem Express, cat. no. HY-N1445), luteolin (Tocris Bioscience, cat. no. 2874), luteolin-7-O-glucuronide (MedChem Express, cat. no. HY-N1463), nobiletin (MedChem Express, cat. no. HY-N015) and rosmarinic acid (MedChem Express, cat. no. HY-N0529).

### SA-β-Gal assay

Senescence was determined by using the SA-β-Gal staining kit (Cell Signaling Technology, cat. no. 9860) according to manufacturer directions. Quantifications were made on at least three images (containing roughly 400–500 cells per field) per experiment by determining the ratio of perinuclear blue-positive to perinuclear blue-negative cells. Fluorescent nuclear staining was performed using Hoechst 33342 (ThermoFisher Scientific, cat. no. H3570). All experiments were quantified by using ImageJ (v.1.54h, National Institutes of Health) software. For experiments with iCMs, analysis for determination of the total number of cells and cells positive for SA-β-Gal was performed with gen5 software (Agilent) in an automated fashion.

For tissue-specific SA-β-Gal assay, immediately after harvesting, kidney samples were stored in optimal cutting temperature compound and snap frozen at −80 °C. The samples were then cut (10 µm) by cryostat. SA-β-Gal staining was performed using the Senescence β-Galactosidase Staining Kit (Cell Signaling Technology) according to the manufacturerʼs instructions. Counterstaining was performed using Eosin (Alcohol-based Diapath, cat. no. C0352).

### Senescence induction in fibroblasts and renal cells

IMR90 fibroblasts were seeded into T75 cm^2^ flasks (Sarstedt) at a density of 5,000 cells cm^−2^ and pretreated with 10 μg ml^−1^ HK or flavonoids (as indicated in Fig. 5b) for 48 h before UV-B irradiation. The culture medium containing the pretreatment was removed, and PBS was added to the cells before UV-B irradiation (200 mJ m^−2^). Irradiation was performed with an Opsytec Dr. Gröbel GmbH Irradiation Chamber. Following irradiation, the prewarmed culture medium containing the respective treatment was added to the flask. Cells were cultured for 5 days to develop a senescent phenotype before seeding in 24-well plates for SA-β-Gal staining.

Doxo was used to induce senescence in WI38 fibroblasts. Cells were pretreated with or without Lut or HK 48 h before the addition of 100 nM Doxo. Following 48 h of exposure to Doxo, the medium was changed to remove Doxo and include either vehicle, Lut or HK for an additional 3 days before seeding in 24-well plates for SA-β-Gal staining.

HK-2 cells were induced to senescence with 100 mg ml^−1^ of albumin for 4 days after pretreatment with Lut or HK for 48 h.

### Induction of senescence in human cardiomyocytes

Senescent phenotype was induced in cardiomyocytes (SenCM) by acute (3 h) exposure to Doxo as previously described^46^. Spontaneously beating iCMs were enzymatically dissociated (Multi Tissue Dissociation Kit 3, Miltenyi Biotec) and plated in Synthemax II-SC Substrate (Corning)-coated wells (5 × 10^4^ cells cm^−2^). After 24 h, iCMs were exposed to 0.2 µM Doxo. After 3 h, cells were washed twice with PBS and placed back in fresh maintenance medium. SenCMs were then treated with HK or Lut and then used for electrophysiological measurement by a multielectrode array (MEA) system or for SA-β-Gal staining.

### Microelectrode arrays

The methodology is described in Supplementary Methods.

### Profiling of secondary metabolites in HK

The methodology is described in Supplementary Methods.

### Determination of luteolin in plasma

The methodology is described in Supplementary Methods.

### Surface plasmon resonance

The interaction between P16 (Sigma-Aldrich, cat. no. SRP3134) and CDK6 (Abcam, cat. no. ab84717) in the presence or not of Luteolin and Fisetin was evaluated at 25 °C by Surface Plasmon Resonance (SPR). In particular, P16 dissolved at a concentration of 200 nM in acetic buffer (pH 4.5) was immobilized on a CM5 chip (Cytiva) through standard amine coupling. Subsequently, increasing concentrations of CDK6 at the dosages of 7.8 nM, 15.6 nM, 31.25 nM, 62.5 nM and 125 nM were injected using a single-cycle kinetics setting. The same experiments were run, including a fixed concertation of 80 mM of Fisetin or Luteolin in the injected samples; 10 mM HEPES pH 7.4, 150 mM NaCl, 3 mM EDTA and 0.005% Tween-20 was used as running buffer. For the experiments run in the presence of Luteolin or Fisetin, 50% of DMSO was added to ensure ligand solubility. All measures were performed with a Biacore 8K instrument, and the Biacore Insight Evaluation Software (v.5.0.18.22102) to analyze the data. To evaluate the reduction of the CDK6-P16 binding in the presence of small molecules, we considered the normalized maximum response unit (RU). This value was obtained by dividing the RU value reached at the end of the injection with the highest concentration of CDK6 by the RU values of the immobilized P16 in the corresponding channel.

### Docking calculations

To obtain atomistic structures of the CDK6–Luteolin complex, we performed docking calculations with Glide using the standard precision mode^49^. The structure of the CDK6–fisetin complex identified from PDB code 1XO2 (ref. ^61^) and prepared by the protein preparation wizard module of the Schrodinger suite was used as the target structure. The grid necessary to perform the docking calculations was centered on the fisetin molecule.

### Proximity ligation assay

WI38 cells were grown on eight-well chamber slides (Nunc, Lab-Tek, cat. no. 177402), treated with DMSO or different concentrations of luteolin, and irradiated with 200 mJ m^−2^ UV-B in an Opsytec Dr. Gröbel GmbH Irradiation chamber. Following an additional 6 h of incubation, cells were washed and fixed in 4% paraformaldehyde. Following three washes with PBS, cells were permeabilized with 0.25% Triton X-100 in PBS for 15 min before blocking buffer supplied by Duolink (Sigma-Aldrich, cat. no. DUO92101) was applied for 1 h. Cells were then incubated overnight at 4 °C with anti-CDK6 (mouse, 1:50; Santa Cruz) and with anti-p16-INK4a (rabbit, 1:50; Proteintech, cat. no. 10883-1-AP). Additional steps such as incubation with secondary probes, washes and mounting was carried out as specified by the manufacturer with Duolink reagents. Images were acquired by an LSM 800 confocal microscope with ZEN v.2.1 software (ZEISS) and the MFI of each cell was analyzed by ImageJ v.1.54h.

### Western blot

Quantum Micro Protein Bicinchoninic Protein Assay (Euroclone) was used to determine the concentration of protein in cell lysates following trypsinization, pelleting and lysis in ice-cold RIPA buffer supplemented with protease (Roche Molecular Biochemicals) and phosphatase inhibitor cocktails (Sigma-Aldrich). Gel electrophoresis was loaded with 15 µg of protein from each sample on a precast 4–12% Bis-Tris ten-well gel (Invitrogen, ThermoFisher Scientific) in an XCell SureLock Mini-Cell system (Invitrogen, ThermoFisher Scientific). Protein was transferred onto a 0.2 µm nitrocellulose membrane (Bio-Rad) with a Trans-Blot Turbo system (Bio-Rad). The membrane was exposed to anti-HSP90 (rabbit, 1:1,000; Cell Signaling Technology, cat. no. 4877), anti-CDK6 (rabbit, 1:1,000; Abcam, cat. no. ab288368), anti-CDK4 (rabbit, 1:1,000; Cell Signaling Technology, cat. no. 12790S), anti-p53 (rabbit, 1:1,000; Cell Signaling Technology, cat. no. 12790), anti-p21 (rabbit, 1:1,000; Cell Signaling Technology, cat. no. 2947), anti-p16 (rabbit, 1:1,000; Cell Signaling Technology, cat. no. 80772), anti-Cyclin D1 (rabbit, 1:1,000; Cell Signaling Technology, cat. no. 2978), anti-GAPDH (mouse, 1:1,000; Santa Cruz, cat. no. SC-365062), anti-Phospho S6 (rabbit, 1:1,000; Cell Signaling Technology, cat. no. 4857S), anti-Phospho-4EBP1 (rabbit, 1:1,000; Cell Signaling Technology, cat. no. 2855), anti-Phospho-eIF2α (rabbit, 1:1000; Cell Signaling Technology, cat. no. 3398T), anti-LC3A/B (rabbit, 1:1000; Cell Signaling Technology, cat. no. 12741T), anti-PGC1alpha (rabbit, 1:1,000; GeneTex, cat. no. GTX135859-25UL), anti-GAPDH (rabbit, 1:1,000; Cell Signaling Technology, cat. no. 5174), anti-Phospho Rb specific for residues Ser807/811 (rabbit, 1:1,000; Cell Signaling Technology, cat. no. 9308) or anti-β-Actin (mouse, 1:1,000; Santa Cruz Biotechnology, cat. no. sc-69879) antibodies after blocking with a 1% bovine serum albumin solution. Following three washes in 1× TBS-T, the membrane was incubated with HRP-conjugated anti-rabbit secondary antibody (goat, 1:10,000; Abcam, cat. no. ab6721) or HRP-conjugated mouse IgG k binding protein (1:5,000; Santa Cruz Biotechnology, cat. no. sc-516102). According to the manufacturerʼs instructions, the membrane was analyzed by adding a chemiluminescent ECL Plus substrate (Euroclone) with a UviTec Alliance Q9 Atom automatic imager. The band intensity was quantified by densitometry with ImageJ processing.

### Reporting summary

Further information on research design is available in the Nature Portfolio Reporting Summary linked to this article.

## Supplementary information

Supplementary Information Supplementary Methods.

Reporting Summary

Supplementary Table 1 Cox proportional hazard regression analysis comparing HK-treated to UT mice in aging. Three models were calculated to determine the influence of animal facility and sex on the effects of treatment. *P* values are two-tailed.

Supplementary Table 2 Transcriptomic signatures MOUSE_ADULT_UP and MOUSE_ADULT_DN. Total RNA was extracted from the gastrocnemius muscle of young (4 months old), old untreated (24 months old) and old treated (24 months old, 4 months of treatment) animals (Young *n* = 8, UT *n* = 10, T *n* = 10). Wald statistical test was used to determine significance of differential expression (*P* values are two-tailed). The same Wald statistics were used to determine enrichments for the MOUSE_ADULT_UP and MOUSE_ADULT_DN gene signatures (*P* values, two-tailed).

Supplementary Table 3 GSEA of manually curated gene sets corresponding to different aging-related pathways in muscle tissue from HK-treated and untreated 24-month-old animals after 4 months of treatment (*n* = 10). Statistical significance was assessed using camera, with a nonparametric, rank-based approach and a presence filter requiring a baseMean ≥50. Normalized enrichment scores were calculated using GSEA preranked.

Supplementary Table 4 Characterization of HK extract. Characterization was performed by UPLC-QTOF, after extracting the sample in methanol. A representative chromatogram is reported in Fig. 5a, while the characterization results are shown in this Table.

Supplementary Table 5 List of primer sequences for RT–qPCR analysis.

## Source data

Source Data Fig. 1 Statistical source data.

Source Data Fig. 3 Statistical source data.

Source Data Fig. 4 Statistical source data.

Source Data Fig. 5 Statistical source data.

Source Data Fig. 6 Statistical source data.

Source Data Fig. 6 Unprocessed western blots.

Source Data Extended Data Figs. 1–5. Statistical source data.

Source Data Extended Data Fig. 3c Unprocessed western blots.

Source Data Extended Data Fig. 5d Unprocessed western blots.

## Data Availability

RNA-seq data that support the findings of this study have been deposited in Gene Expression Omnibus (GEO) under the accession code GSE266005. The crystallographic data used in this study are available from the Protein Data Bank under accession code 1XO2. Statistical source data for Figs. 1, 3–6 and Extended Data Figs. 1–5 are provided with this paper. All other data supporting the findings of this study are available from the corresponding author upon reasonable request.
