# Supplementary material for: Targeting senescence induced by age or chemotherapy with a polyphenol-rich natural extract improves longevity and healthspan in mice
Source: Nat Aging. 2024 Jul 1;4(9):1231–48. doi: 10.1038/s43587-024-00663-7 (PMC11408255; doi:10.1038/s43587-024-00663-7)
Supplement: Supplementary file 1 — Supplementary Methods. [file 43587_2024_663_MOESM1_ESM.pdf]

nature aging

Supplementary information

https://doi.org/10.1038/s43587-024-00663-7

Targeting senescence induced by age or chemotherapy with a polyphenol-rich natural extract improves longevity and healthspan in mice

In the format provided by the authors and unedited

SUPPLEMENTARY METHODS

Histological evaluation of articulations

After micro-CT analysis, specimens were decalcified, paraffin-embedded, and sectioned in 5µm thick slices. Sections were stained with hematoxylin, eosin (H&E), or safranin O/fast green to evaluate cartilage damage and identify proteoglycans. Slides were observed, and digital images were collected using a LEICA DM750 microscope and ICC50 camera. The severity of OA was scored by two blinded independent investigators using the Osteoarthritis Research Society International (OARSI) grade system. Proteoglycan content was scored in safranin O/fast green stained slides on a scale of 0–4 where 0=no loss, 1=minimal loss, 2=mild loss, 3=moderate loss, and 4=marked loss of proteoglycan staining.

Micro-computed tomography analysis

Each sample was placed in a cylindrical polyethylene container and analyzed by an *ex vivo* high-resolution Micro-CT 1275 (Bruker, Kontich, Belgium). The following Micro-CT parameters were applied: 65 Kv, 80 µA, 1 mm aluminum filter, and 21 µm voxel size. The acquired raw data were reconstructed and analyzed with the N-Recon version 2.0 and CT-An version 1.18 software, respectively (Bruker microCT, Kontich, Belgium). To compute the bone mineral density (mg/mm^3), one sample of saline solution and two samples of CaHA of 0.25 and 0.75 g/cm^3 were acquired and reconstructed by applying the same parameters. For each sample, cortical bone volume (%) and bone mineral density (mg/mm^3) of the bone marrow were computed.

Microelectrode arrays (MEA)

Field potentials of spontaneously beating clusters of iCMs and SenCMs were recorded at 37°C using a 60MEA100/10iR-Ti-gr 64-electrode Microelectrode Arrays (MEA, Multi Channel Systems). Colony-matched iCMs and SenCM were seeded in the Syntemax-coated MEA chambers (volume 500 µl). The latter were performed in a maintenance medium or in the presence of Haenkenium (100 µg/ml). Measurements were taken 10 minutes after signals reached a steady

state value for each time point. The duration of field potentials, reflecting the electrical systole, was measured from the onset of the sharp positive deflection to the peak of the secondary slow deflection. This measurement is representative of the electrocardiographic QT interval. The rate-corrected QT intervals (QTc) were calculated by applying Bazett's correction ($QTc = QT/\sqrt{RR}$). MEA data analysis was performed with MC Rack version 4.6.2, MC Data Tool version 2.6.15 (both by Multi Channel Systems), and Clampfit 10.7 (Molecular Devices).

Profiling of secondary metabolites in Haenkenium

Chemical profiling of HK extract was performed by using UPLC-QTOF-MS. Samples were prepared by suspending 150 mg of dried plant material in 1 mL of methanol and sonicating for 20 min. Afterward, the mixtures were centrifuged at 13,000 rpm for 10 min, and the supernatants were used for analysis. This latter was carried out using Waters Acquity UPLC system coupled to a Waters Xevo G2 QTOF MS detector, operating in ESI (-) mode. For the chromatographic separation, an Agilent Eclipse plus C18 column (2.1x50 mm, 1.8 µm) was used as the stationary phase, and a gradient mixture of acetonitrile (A) and 0.1% formic acid in water (B) was used as mobile phase. The gradient was: 0 min, 2% A; 1 min, 2% A; 11 min, 85% A; 16 min, 100% A; 20 min, 100% A; 21 min, 2% A and isocratic up to 24 min. The flow rate was 0.30 mL/min. MS parameters were as follows: sampling cone voltage, 40 V; source offset, 80 V; capillary voltage, 3,500 V; nebulizer gas (N 2) flow rate, 800 L/h; desolvation temperature, 450°C. The mass accuracy and reproducibility were maintained by infusing lock mass (leucine-enkephalin [M–H] − = 554.2620 m/z) through Lockspray at a flow rate of 20 µL/min. Centroid data were collected in the m/z range of 50 –1,200, and the m/z values were automatically corrected during acquisition using lock mass. MS e data were also collected to obtain information about the fragmentation of detected compounds, applying a collision voltage of 30 V. Accurate m/z values of parent ions and fragments were then used to perform tentative identification of metabolites by comparison with open source databases (HMDB: https://hmdb.ca/; Metlin: https://metlin.scripps.edu/) and available literature. The quantification of flavonoids, phenolic acids and triterpenes was done using calibration curves built from a solution of reference standards at increasing concentrations, namely rutin, chlorogenic acid, and oleanolic acid, respectively. Calibration curves were: y = 5549.8x +

2629 (rutin; R2 = 0.998); y = 2139.2x – 3293.1 (chlorogenic acid; R2 = 0.998); y = 4434.4x + 1589.9 (oleanolic acid; R2 = 0.999).

Determination of luteolin in plasma

Plasma samples were treated with a solution formed by Acetonitrile 5% formic acid with 760ng/mL of ISTD (benzanilide). Plasma samples of 20 to 30 µL were added with 150 µL of solution for the LC-MS/MS analysis. The analysis was performed on a Waters LC system coupled with triple quadrupole Mass spectrometer (Varian) model 320 equipped with Electrospray Ion Source. As stationary phase an Agilent Eclipse XDB C18 2.1 x 150 mm (3.5 micron). As mobile phases water 0.1% formic acid (A) and acetonitrile (B) were used. Gradient was started with 90% A and in 10 minutes went to 15% A, then at 15 minutes 5% A and stayed isocratic up to 16 minutes, before back to initial for re-equilibration. Recorded flow rate was 200 µL/min. For the Electrospray the Needle potential was 4900 V and drying gas was set to 20 psi while nebulizer was 50 psi, spray shield was 450. The ISTD was monitored in positive mode following the transition 198>105 and the retention time was 11.5 minutes. Luteolin was monitored in negative mode following the transition of 285>150.6 and 285>132.5 using this latter as quantifier, retention time was 9.9 minutes. The calibration curve was obtained mixing solution of luteolin and ISTD in different ratios and acquiring the chromatograms. The LOQ was set as 0.5 ng/mL.
