# Supplementary material for: Targeting senescence induced by age or chemotherapy with a polyphenol-rich natural extract improves longevity and healthspan in mice
Source: Nat Aging. 2024 Jul 1;4(9):1231–48. doi: 10.1038/s43587-024-00663-7 (PMC11408255; doi:10.1038/s43587-024-00663-7)
Supplement: Supplementary file 2 — Reporting Summary [file 43587_2024_663_MOESM2_ESM.pdf]

nature portfolio

Corresponding author(s): Andrea Alimonti, Monica Montopoli

Last updated by author(s): Apr 27, 2024

Reporting Summary

Nature Portfolio wishes to improve the reproducibility of the work that we publish. This form provides structure for consistency and transparency in reporting. For further information on Nature Portfolio policies, see our Editorial Policies and the Editorial Policy Checklist.

Statistics

For all statistical analyses, confirm that the following items are present in the figure legend, table legend, main text, or Methods section.

n/a	Confirmed	
☐	☒	The exact sample size (*n*) for each experimental group/condition, given as a discrete number and unit of measurement
☒	☐	A statement on whether measurements were taken from distinct samples or whether the same sample was measured repeatedly
☐	☒	The statistical test(s) used AND whether they are one- or two-sided<br>*Only common tests should be described solely by name; describe more complex techniques in the Methods section.*
☒	☐	A description of all covariates tested
☐	☒	A description of any assumptions or corrections, such as tests of normality and adjustment for multiple comparisons
☐	☒	A full description of the statistical parameters including central tendency (e.g. means) or other basic estimates (e.g. regression coefficient) AND variation (e.g. standard deviation) or associated estimates of uncertainty (e.g. confidence intervals)
☐	☒	For null hypothesis testing, the test statistic (e.g. *F*, *t*, *r*) with confidence intervals, effect sizes, degrees of freedom and *P* value noted<br>*Give P values as exact values whenever suitable.*
☒	☐	For Bayesian analysis, information on the choice of priors and Markov chain Monte Carlo settings
☒	☐	For hierarchical and complex designs, identification of the appropriate level for tests and full reporting of outcomes
☒	☐	Estimates of effect sizes (e.g. Cohen's *d*, Pearson's *r*), indicating how they were calculated

Our web collection on statistics for biologists contains articles on many of the points above.

Software and code

Policy information about availability of computer code

Data collection	Immunohistochemistry: Aperio ScanScope (Leica Biosystem). Beta-galactosidase image acquisistion: Nexcope microscope. Western blot and Proteome Profiler acquisition: Uvitec Alliance Atom Q9. Real-time PCR: QuantStudio 5 (Applied Biosystem). Body composition: EchoMRI™-100 (EchoMRI LLC). Bone analysis: Micro-CT 1275 (Bruker). Grip strength: BIOSEB, BIO-GS3. Immunofluorescence: DM6B (Leica biosystem). In vivo luminescence detection: IVIS imaging system (IVIS spectrum, Perkin Elmer). QT intervals of cardiac myocytes: 60MEA100/10iR-Ti-gr 64-electrode (MEA, Multi Channel Systems). Surface plasmon resonance: Biacore 8K instrument. UPLC-QTOF-MS: Waters Acquity UPLC system coupled to a Waters Xevo G2 QTOF MS detector. Proximity Ligation Assay: Zeiss LSM 800 Confocal microscope.
Data analysis	Microsoft Excel 2018. GraphPad Prism v10.2.0. ImageScope v12.3.2.8013 (Leica Biosystem). FastQC v0.11.8. ImageJ 1.54h. QuantStudio 5 v1.4.3 (Applied Biosystems). MC Rack v4.6.2 (Multi Channel System). MC Data Tool v2.6.15 (Multi Channel Systems). Clampfit 10.7 (Molecular Devices). CTAn v1.18 (Bruker). N-Recon v 2.0 (Bruker). Biacore Insight Evaluation Software v5.0.18.22102 (Cytiva).

For manuscripts utilizing custom algorithms or software that are central to the research but not yet described in published literature, software must be made available to editors and reviewers. We strongly encourage code deposition in a community repository (e.g. GitHub). See the Nature Portfolio guidelines for submitting code & software for further information.

Data

Policy information about availability of data

All manuscripts must include a data availability statement. This statement should provide the following information, where applicable:

- Accession codes, unique identifiers, or web links for publicly available datasets
- A description of any restrictions on data availability
- For clinical datasets or third party data, please ensure that the statement adheres to our policy

The authors declare that all data supporting the findings of this study are available within the paper and its Extended Information files. RNA-seq data that support the findings of this study have been deposited in Gene Expression Omnibus (GEO) under the accession code GSE266005. The crystallographic data used in this study are available from the Protein Data Bank under accession code 1XO2 [https://www.rcsb.org/structure/1xo2]. Source Data for Figs. 1,3-6 and Extended Data Figs. 1-5 have been provided as Source Data files. All other data supporting the findings of this study are available from the corresponding author upon reasonable request.

Research involving human participants, their data, or biological material

Policy information about studies with human participants or human data. See also policy information about sex, gender (identity/presentation), and sexual orientation and race, ethnicity and racism.

Reporting on sex and gender	The study did not involve human participants.
Reporting on race, ethnicity, or other socially relevant groupings	The study did not involve human participants.
Population characteristics	The study did not involve human participants.
Recruitment	The study did not involve human participants.
Ethics oversight	The study did not involve human participants.

Note that full information on the approval of the study protocol must also be provided in the manuscript.

Field-specific reporting

Please select the one below that is the best fit for your research. If you are not sure, read the appropriate sections before making your selection.

☒ Life sciences ☐ Behavioural & social sciences ☐ Ecological, evolutionary & environmental sciences

For a reference copy of the document with all sections, see nature.com/documents/nr-reporting-summary-flat.pdf

Life sciences study design

All studies must disclose on these points even when the disclosure is negative.

Sample size	No statistical method was used to predetermine sample size.<br>For animal studies, sample size was defined on the basis of literature data (Baker et al., Nature 2016) and on past experience with the models.<br>For ethical reasons, the minimum number of animals necessary to achieve the scientific objectives was used.<br>For other experiments, group sizes were determined based on the results of preliminary experiments, taking in consideration the means of the target values between the experimental group and the control group, the standard error and the statistical analysis used.
Data exclusions	Data exclusions Grubb's test was applied to exclude outliers. All samples meeting proper experimental conditions were included in the analysis; samples were excluded in reason of compromised specimen integrity/quality, that would have negatively affected the analysis.
Replication	Experiments were replicated several times with reproducible results as indicated in figure legends.
Randomization	Cell lines and animals were allocated randomly to each treatment group. Different treatment groups were processed identically, and animals in different treatment groups were exposed to the same environment.
Blinding	For in vivo studies, blinding was not possible as the presence/absence of the treatment in the drinking water was visible. However, lifespan studies were performed in different animal facilities and independently confirmed by at least 2 investigators involved in the study.<br>Analysis performed by pathologists including kidney H&E and kidney p16 IHC were performed blind. For all the other experiments blinding was not possible as the same operator was responsible for treatment and analysis. However, critical experiments (i.e. SA-b-gal measurement) have been independently performed by at least 2 different investigators.

Reporting for specific materials, systems and methods

We require information from authors about some types of materials, experimental systems and methods used in many studies. Here, indicate whether each material, system or method listed is relevant to your study. If you are not sure if a list item applies to your research, read the appropriate section before selecting a response.

Materials & experimental systems

n/a	Involved in the study
☐	☒ Antibodies
☐	☒ Eukaryotic cell lines
☒	☐ Palaeontology and archaeology
☐	☒ Animals and other organisms
☒	☐ Clinical data
☒	☐ Dual use research of concern
☐	☒ Plants

Methods

n/a	Involved in the study
☒	☐ ChIP-seq
☒	☐ Flow cytometry
☒	☐ MRI-based neuroimaging

Antibodies

Antibodies used

For immunohistochemistry: anti-p16 (rabbit, 1:1000, Abcam, ab211542); anti-p27 (rabbit, 1:1000, Abcam, ab32034); anti-γH2AX (rabbit, 1:1000, Cell Signaling, 9718); anti-53BP1 (rabbit, 1:1000, Abcam, ab21083); biotinylated goat anti-rabbit IgG antibody (Vector Laboratories BP-9100).
For immunofluorescence: anti-dystrophin (Thermo Fisher Scientific #PA5-32388, lot GR3443485-4); goat anti-rabbit-CY3 (Thermo Fisher Scientific #A10520, lot 122871).
For western blot: anti-HSP90 (rabbit, 1:1000; Cell Signaling Technology #4877), anti-CDK6 (rabbit, 1:1000; Abcam ab288368), anti-CDK4 (rabbit, 1:1000; Cell Signaling Technology #12790S), anti-p53 (rabbit, 1:1000; Cell Signaling Technology #12790), anti-p21 (rabbit, 1:1000; Cell Signaling Technology #2947), anti-p16 (rabbit, 1:1000; Cell Signaling Technology #80772), anti-Cyclin D1 (rabbit, 1:1000; Cell Signaling Technology #2978), anti-GAPDH (mouse, 1:1000; Santa Cruz SC-365062), anti-Phospho S6 (rabbit, 1:1000; Cell Signaling Technology #4857S), anti-Phospho-4E-BP1 (rabbit, 1:1000; Cell Signaling Technology #2855), anti-Phospho-eIF2α (rabbit, 1:1000; Cell Signaling Technology #3398T), anti-LC3A/B (rabbit, 1:1000; Cell Signaling Technology #12741T), anti-PGC1alpha (rabbit, 1:1000; GeneTex GTX135859-25UL), anti-GAPDH (rabbit, 1:1000; Cell Signaling Technology #5174), anti-Phospho Rb specific for residues Ser807/811 (rabbit, 1:1000; Cell Signaling Technology #9308), anti-β-Actin (mouse, 1:1000; Santa Cruz Biotechnology sc-69879); (HRP)-conjugated anti-rabbit secondary antibody (goat, 1:10000; Abcam ab6721); HRP-conjugated mouse IgG k binding protein (1:5000; Santa Cruz Biotechnology sc-516102). .

Validation

The validation of each primary antibody for the species and applications is available from the manufacturer's websites. For immunofluorescence and Western Blot analysis, standardized dilution previously set up in the laboratory or by the investigator was used. For murine IHC, preliminary tests with increasing dilutions and different unmasking pH solutions were performed.

Eukaryotic cell lines

Policy information about cell lines and Sex and Gender in Research

Cell line source(s)

IMR-90 (CCL-186™, female), WI-38 (CCL7™, female), and HK-2 (CRL-2190™, male) cell lines were purchased from ATCC. Human cardiomyocytes were derived from induced-pluripotent-stem-cell (iPS) using a cell-line previously generated and established in the laboratory.

Authentication

The used cell lines were not authenticated.

Mycoplasma contamination

Mycoplasma contamination was routinely tested by PCR (N-GARDE Mycoplasma PCR Reagent Set, Euroclone #EMK090020). Only mycoplasma-negative cells were used for the experiments.

Commonly misidentified lines
(See ICLAC register)

No commonly misidentified cell lines were used in the study.

Animals and other research organisms

Policy information about studies involving animals; ARRIVE guidelines recommended for reporting animal research, and Sex and Gender in Research

Laboratory animals

Aging experiments were performed using C57BL/6 mice purchased from Charles River at the age of 78 weeks or with animals bred and aged in the animal facility of the Pharmacy Department (University of Padova). Animal treatment was started at the age of 20 months.
Doxorubicin injection was performed on C57BL/6 and p16LUC mice. p16LUC mice (B6.Cg-Cdkn2atm3.1Nesh Tyr c-2J/Nci) from Dr. Norman Sharpless were obtained from NCI Mouse Repository and were maintained under specific pathogen-free conditions in the IRB animal facility of the Università della Svizzera italiana (Bellinzona, Switzerland). Animal treatment was started at the age of 10 weeks.
Mice were maintained at room temperature (20-22°C), with humidity at 55 ± 10 %, and exposed to a 12-hour daylight cycle.

Wild animals

The study did not involve wild animals.

Reporting on sex	Both male and female animals were included in the study. Disaggregated data for overall survival are provided for both sexes.
Field-collected samples	The study did not involve samples collected from the field.
Ethics oversight	Experiments were performed under Italian national and EU directives (2010/63/EU) for animal research with protocols approved by Institute Ethical Committee and the Italian Ministry of Health (672/2019-PR and 336/2020-PR), or according to Swiss state guidelines and approved by the local ethics committee ("Dipartimento della Sanità e Socialità, Esperimenti su animali" Canton Ticino), authorization numbers 30275 and 34293.

Note that full information on the approval of the study protocol must also be provided in the manuscript.

Dual use research of concern

Policy information about dual use research of concern

Hazards

Could the accidental, deliberate or reckless misuse of agents or technologies generated in the work, or the application of information presented in the manuscript, pose a threat to:

No	Yes	
☒	☐	Public health
☒	☐	National security
☒	☐	Crops and/or livestock
☒	☐	Ecosystems
☒	☐	Any other significant area

Experiments of concern

Does the work involve any of these experiments of concern:

No	Yes	
☒	☐	Demonstrate how to render a vaccine ineffective
☒	☐	Confer resistance to therapeutically useful antibiotics or antiviral agents
☒	☐	Enhance the virulence of a pathogen or render a nonpathogen virulent
☒	☐	Increase transmissibility of a pathogen
☒	☐	Alter the host range of a pathogen
☒	☐	Enable evasion of diagnostic/detection modalities
☒	☐	Enable the weaponization of a biological agent or toxin
☒	☐	Any other potentially harmful combination of experiments and agents
