# Supplementary material for: Targeting senescence induced by age or chemotherapy with a polyphenol-rich natural extract improves longevity and healthspan in mice
Source: Nat Aging. 2024 Jul 1;4(9):1231–48. doi: 10.1038/s43587-024-00663-7 (PMC11408255; doi:10.1038/s43587-024-00663-7)
Supplement: Supplementary file 3 — Cox proportional hazard regression analysis comparing HK-treated to UT mice in aging. Three models were calculated to determine the influence of animal facility and sex on the effects of treatment. P values are two-tailed. [file 43587_2024_663_MOESM3_ESM.pdf]

Table S1 Cox proportional hazard ratios comparing HK-treated to UT mice in aging. Three models were calculated to determine the effect of facility and sex on the effects of treatment.

**Model 1: Unadjusted**					
		**P value**	**Hazard Ratio**	**95% CL**	
**Treatment**	**HK vs UT**	0.0064	0.54	0.347	0.841
**Model 2: stratified by facility**					
**Treatment**	**HK vs UT**	0.0115	0.559	0.356	0.878
**Model 3: stratified by facility & adjusted by sex**					
**Treatment**	**HK vs UT**	0.0226	0.577	0.359	0.926
**Sex**	**Female**	0.6525	0.898	0.561	1.437
