# Supplementary material for: Targeting senescence induced by age or chemotherapy with a polyphenol-rich natural extract improves longevity and healthspan in mice
Source: Nat Aging. 2024 Jul 1;4(9):1231–48. doi: 10.1038/s43587-024-00663-7 (PMC11408255; doi:10.1038/s43587-024-00663-7)
Supplement: Supplementary file 6 — Characterization of HK extract. Characterization was performed by UPLC-QTOF, after extracting the sample in methanol. A representative chromatogram is reported in Fig. 5a, while the characterization results are shown in this Table. [file 43587_2024_663_MOESM6_ESM.pdf]

Supplementary Table 4

CHARACTERIZATION OF HAENKENIUM EXTRACT

Characterization was performed by UPLC-QTOF, after extracting the sample in methanol. A representative chromatogram is reported in Figure 5a, while the characterization results are shown in this Table.

RT (min)	m/z	Fragments (MS/MS)	Identification	Ion type	% w/w *
9.2749	637.1194	285.0399 175.0387 151.0387	Luteolin diglucuronide	$[M-H]^-$	0.55
7.9191	315.0499	151.0288	Isorhamnetin	$[M-H]^-$	0.48
7.2652	401.1238	211.0828	Nobiletin	$[M-H]^-$	0.38
6.8429	461.0719	285.0396 151.0024 133.0283	Luteolin glucuronide	$[M-H]^-$	0.59
5.8229	593.1515	473.1082 383.0767 353.0662	Elatin	$[M-H]^-$	0.32
8.6852	329.0661	314.0421 299.0188	3,7-Dimethylquercetin	$[M-H]^-$	0.21
8.7185	577.1924	285.0393	Prenyleriodictyol	$[M-H]^-$	0.21
8.4595	285.0397	175.0388 151.0027 133.0285 107.1270	Luteolin	$[M-H]^-$	0.15
9.1078	591.1715	301.1163	Hesperetin 7-neohesperidoside	$[M-H_2O-H]^-$	0.11
8.0571	533.1647	371.1120	Phellatin	$[M-H]^-$	0.09
6.9794	463.0874	301.0336	Quercetin-glucoside (isoquercetin)	$[M-H]^-$	0.07
9.9239	299.0551	284.0316 256.0352	Diosmetin	$[M-H]^-$	0.05
9.2643	593.1502	285.0387	Luteolin-7-O-rutinoside	$[M-H]^-$	0.04
5.5006	609.1447	301.0269	Rutin	$[M-H]^-$	0.02
9.2690	285.0399	255.0289 227.0346	Kaempferol	$[M-H]^-$	0.02
			**Total identified flavonoids**		**3.29**
7.8315	403.1399	357.1332 163.0388 151.0389	Pinoresinol #	$[M+FA-H]^-$	7.29
7.3855	359.0767	197.0450 179.0343 161.0239	Rosmarinic acid	$[M-H]^-$	4.92

7.3855	719.1615	359.0766 161.0236	Sagerinic acid	$[M-H]^-$	2.58
8.0107	553.2286	521.2021 507.2228 465.1761 419.1968	Ssioriside	$[M-H]^-$	1.9
8.7593	515.1188	353.0874	Di-caffeoylquinic acid	$[M-H]^-$	0.92
7.9846	627.2288	581.2233 401.1595 59.0123	Lyoniresinol 9-glucoside #	$[M+FA-H]^-$	0.58
8.5969	567.1147	NA	Chrysophanol 8-(6-galloylglucoside)	$[M-H]^-$	0.58
6.5666	717.1464	537.2167 519.0921 475.1023	Salvianolic acid L	$[M-H]^-$	0.64
8.3924	515.1184	179.0339	Dicaffeoylquinic acid	$[M-H_2O-H]^-$	0.92
6.7077	373.1285	NA	8-Hydroxypinoresinol #	$[M-H]^-$	0.44
7.3945	161.0235	179.0338	Caffeic acid	$[M-H_2O-H]^-$	0.43
6.8777	555.1128	313.0718	Salvianolic acid K	$[M-H]^-$	0.07
			**Total identified phenols and lignans #**		**21.26**
5.6685	387.1651	225.0790 207.1017	beta-D-Glucopyranosyl-11-hydroxyjasmonic acid §	$[M-H]^-$	3.92
13.6175	571.2884	409.2351	Phosphatidylinositol §	$[M-H]^-$	0.19
15.8469	455.3522	437.3423 411.3579	Ursolic acid	$[M-H]^-$	0.24
9.5309	347.1860	329.1734 317.1751	2α,16-Dihydroxy-4β-carboxy-*O*-β-D-glucopyranosyl-19-nor-totarol	$[M-H]^-$	0.13
13.9808	455.3528	NA	Oleanolic acid	$[M-H]^-$	0.16
8.7593	389.1596	NA	Rosmic acid	$[M-H]^-$	0.1
11.5745	331.1911	287.2020	Carnosic acid	$[M-H]^-$	0.05
15.7777	471.3474	453.3380	Corosolic acid	$[M-H]^-$	0.05
12.3086	487.3429	NA	Salvin A	$[M-H]^-$	0.05
16.7308	730.5480	568.4941	Araliacerebroside §	$[M-H]^-$	0.07
16.0299	461.3632	415.1462 149.0967	beta-Tocopherol	$[M+FA-H]^-$	0.05
12.4451	471.3476	NA	Maslinic acid	$[M-H]^-$	0.05
6.4211	369.0827	207.0285	5-Hydroxy-6-methoxycoumarin 7-glucoside §	$[M-H]^-$	0.02
			**Total identified terpenes and others §**		**5.08**

* Flavonoids amounts are expressed as rutin equivalents; phenols as chlorogenic acid equivalents; terpenes as oleanolic acid equivalents; glycerides and fatty acid derivatives as lecithin equivalents; coumarins as coumarin equivalents.

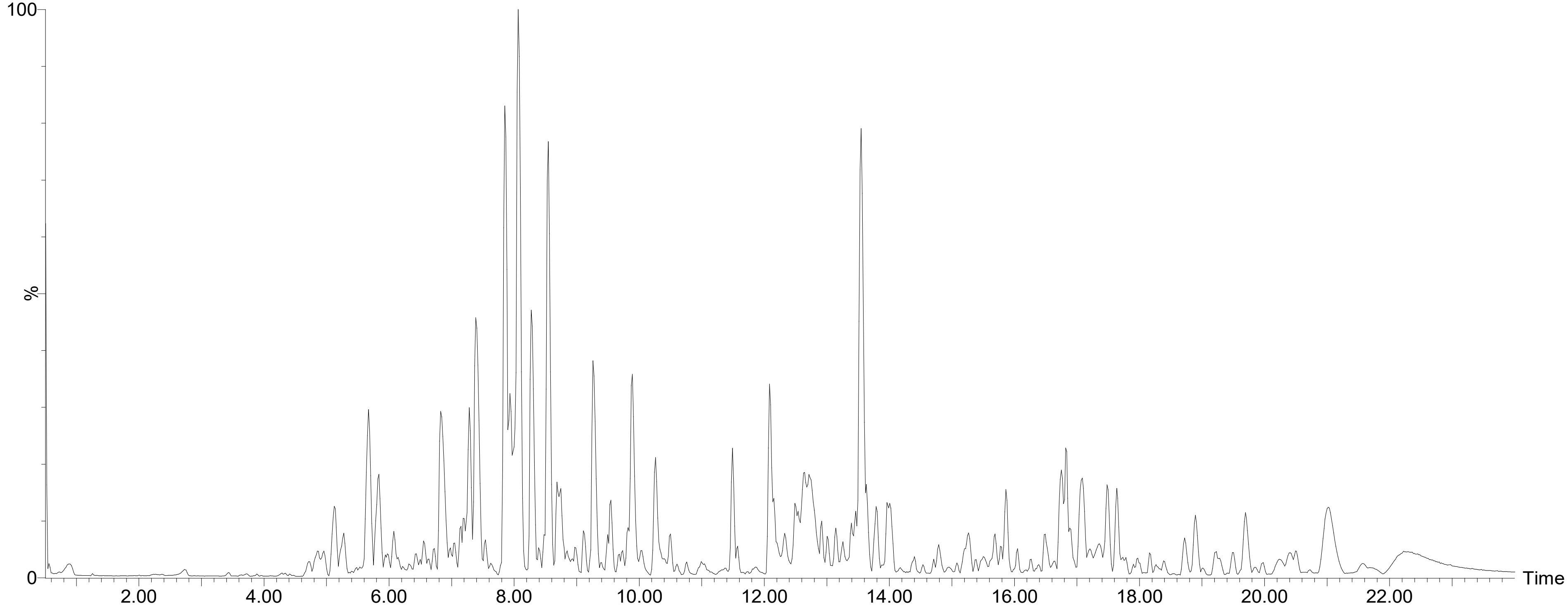

100
%
0
2.00
4.00
6.00
8.00
10.00
12.00
14.00
16.00
18.00
20.00
22.00
Time
