# Supplementary material for: Targeting senescence induced by age or chemotherapy with a polyphenol-rich natural extract improves longevity and healthspan in mice
Source: Nat Aging. 2024 Jul 1;4(9):1231–48. doi: 10.1038/s43587-024-00663-7 (PMC11408255; doi:10.1038/s43587-024-00663-7)
Supplement: Supplementary file 7 — List of primer sequences for RT–qPCR analysis. [file 43587_2024_663_MOESM7_ESM.pdf]

Supplementary Table 5 - List of primer sequences for q-RT-PCR analysis

mRNA	Primer
Mus musculus *Col1a1*	Sense: 5'-GCTCCTCTTAGGGGCCACT-3'
	Anti-sense: 5'-CCACGTCTCACCATTGGGG-3'
Mus musculus *Col3a1*	Sense: 5'-ACGTAGATGAATTGGGATGCAG-3'
	Anti-sense: 5'-GGGTTGGGGCAGTCTAGTG-3'
Mus musculus *Fn1*	Sense: 5'-ATGTGGACCCCTCCTGATAGT-3'
	Anti-sense: 5'-GCCCAGTGATTTCAGCAAAGG-3'
Mus musculus *Cdk1na*	Sense: 5'-CCCCCAATCGCAAGGATTCTT-3'
	Anti-sense: 5'-CTTGGTTCGGTGGGTCTGTC-3'
Mus musculus *Tp53*	Sense: 5'-CTCTCCCCCGCAAAAGAAAAA-3'
	Anti-sense: 5'-CGGAACATCTCGAAGCGTTTA-3'
Mus musculus *Gapdh*	Sense: 5'- AGGTCGGTGTGAACGGATTTG-3'
	Anti-sense: 5'- TGTAGACCATGTAGTTGAGGT-3'
Homo sapiens *CDK1NA*	Sense: 5'- CACCTCACCTGCTCTGCTGC -3'
	Anti-sense: 5'- GCTGGTCTGCCGCCGTTTT -3'
Homo sapiens *RPL27*	Sense: 5'- TGGTAGGGCCGGGTGGTTGC -3'
	Anti-sense: 5'- ACTTTGCGGGGGTAGCGGTC -3'
Homo sapiens *GAPDH*	Sense: 5'- TGCACCACCAACTGCTTAGC -3'
	Anti-sense: 5'- GGCATGGACTGTGGTCATGAG -3'
