# Supplementary material for: Targeting senescence induced by age or chemotherapy with a polyphenol-rich natural extract improves longevity and healthspan in mice
Source: Nat Aging. 2024 Jul 1;4(9):1231–48. doi: 10.1038/s43587-024-00663-7 (PMC11408255; doi:10.1038/s43587-024-00663-7)
Supplement: Supplementary file 13 — Unprocessed western blots. [file 43587_2024_663_MOESM13_ESM.pdf]

Ponceau

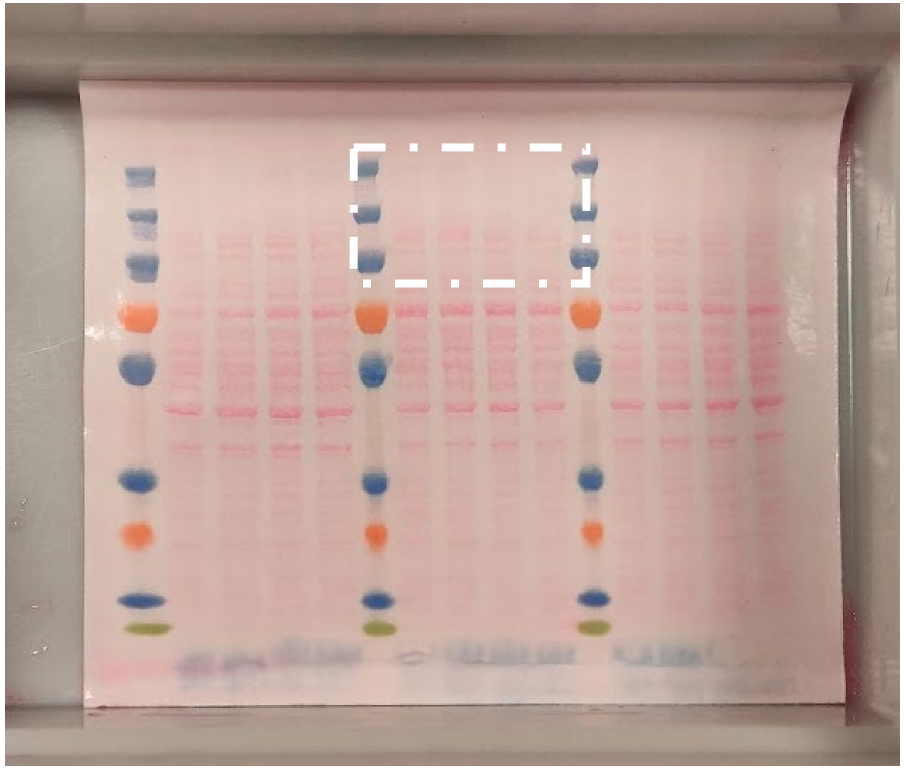

HSP90

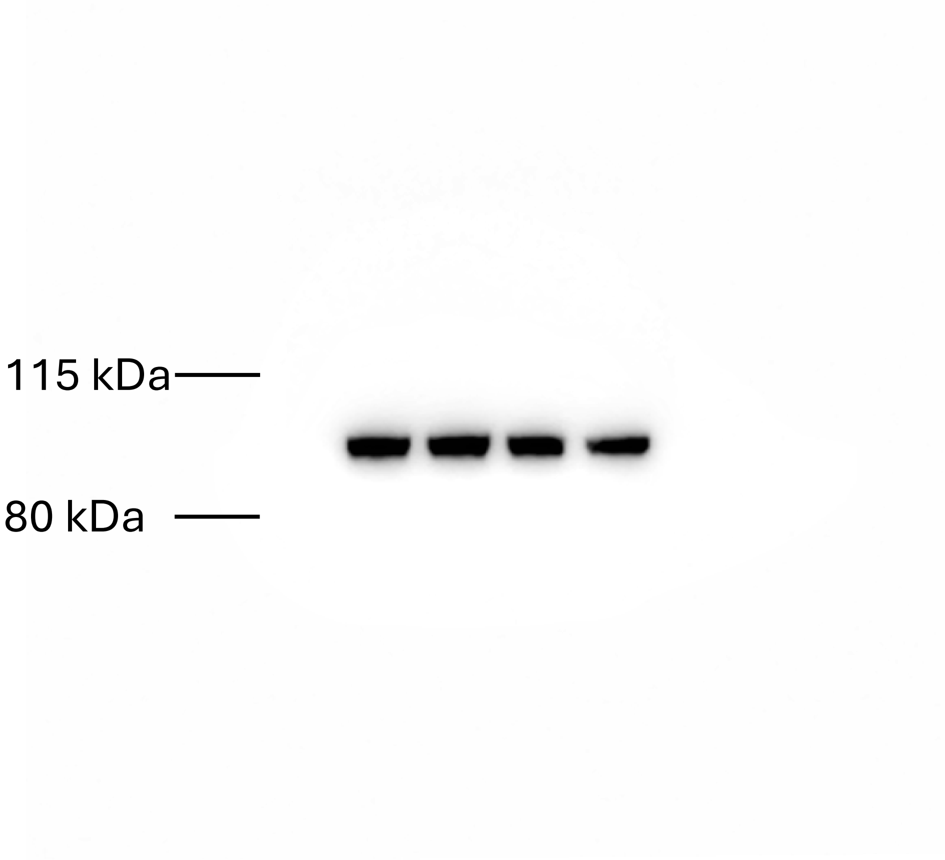

Ponceau

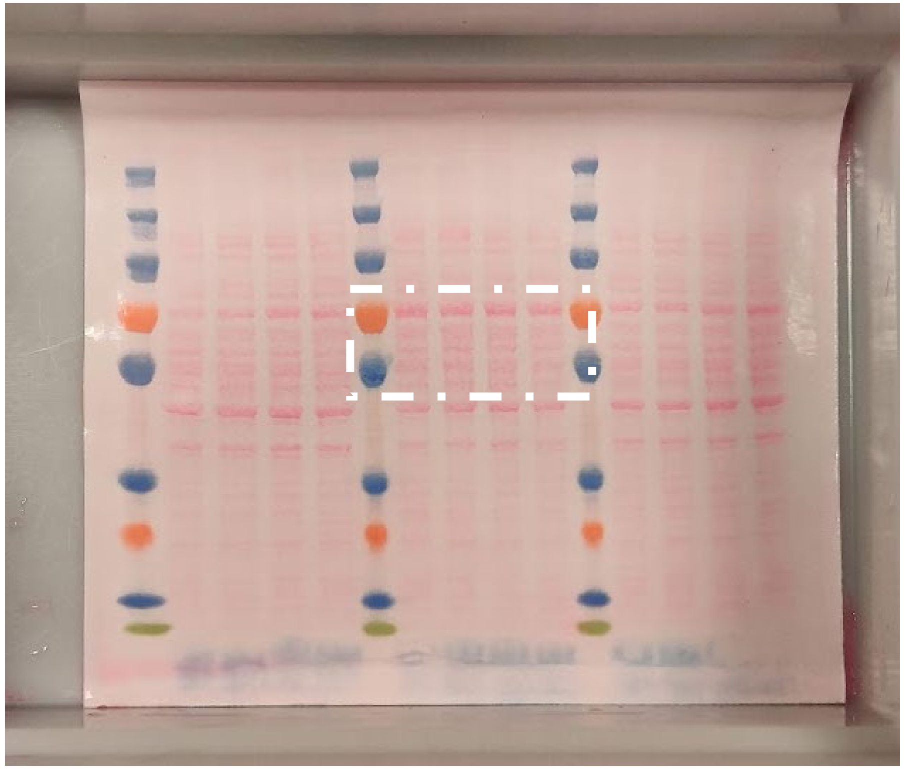

p53

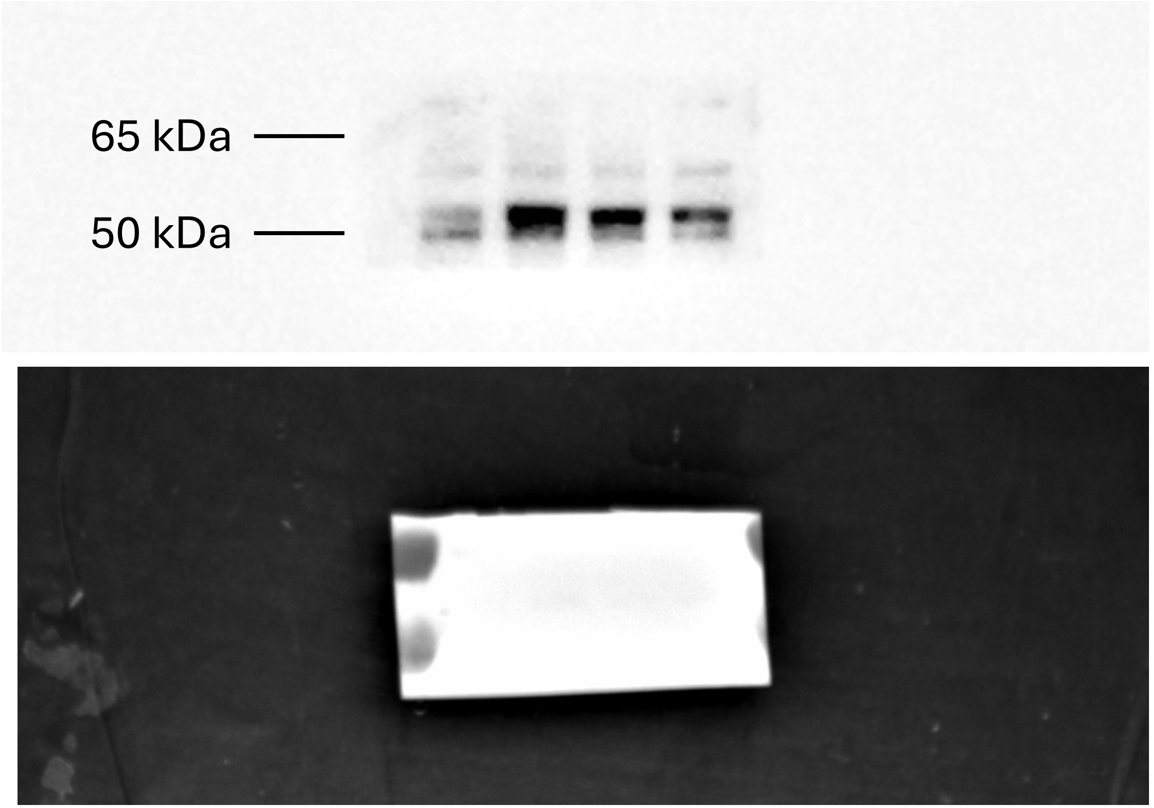

Ponceau

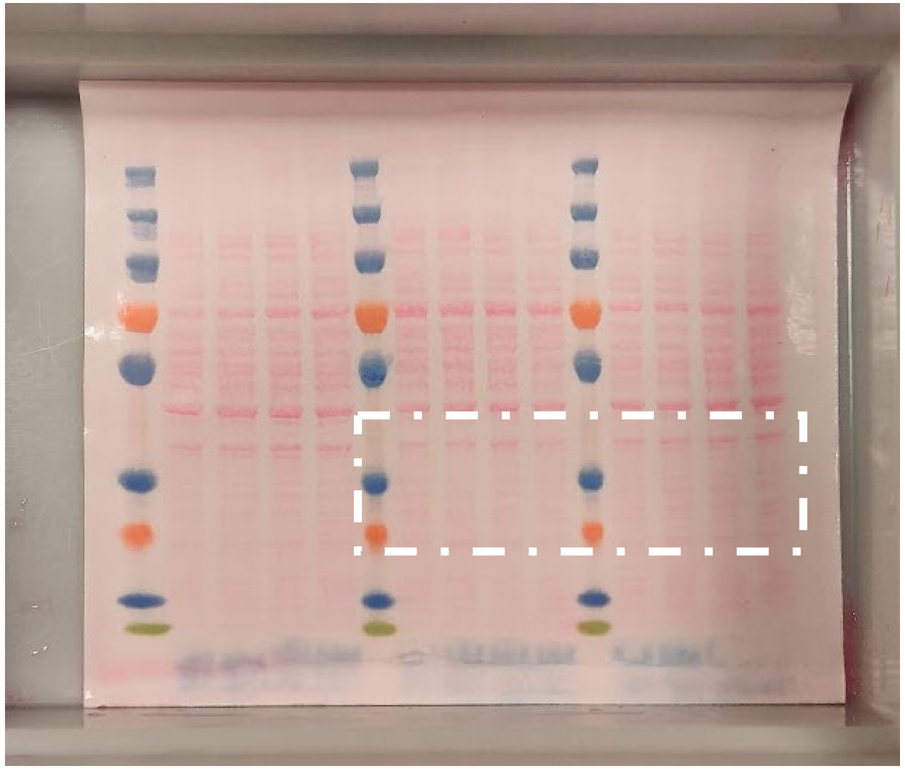

CDK4

30 kDa

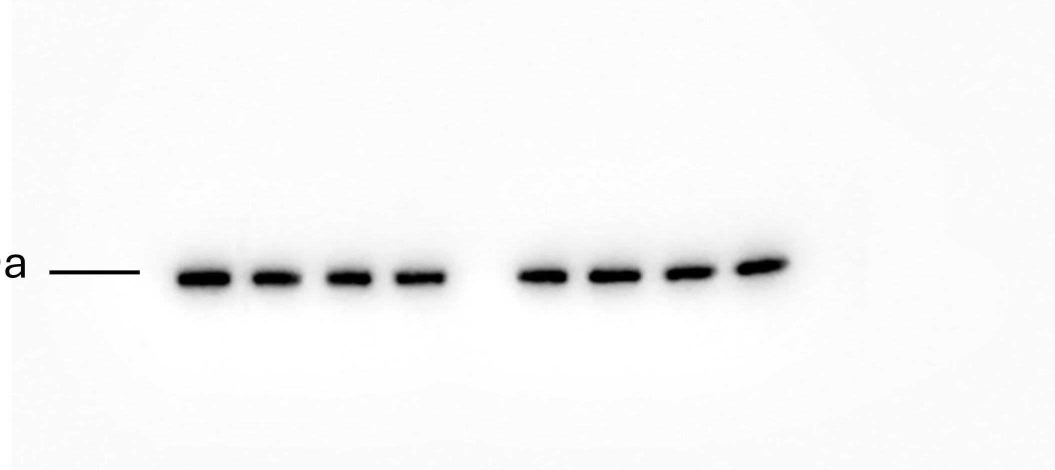

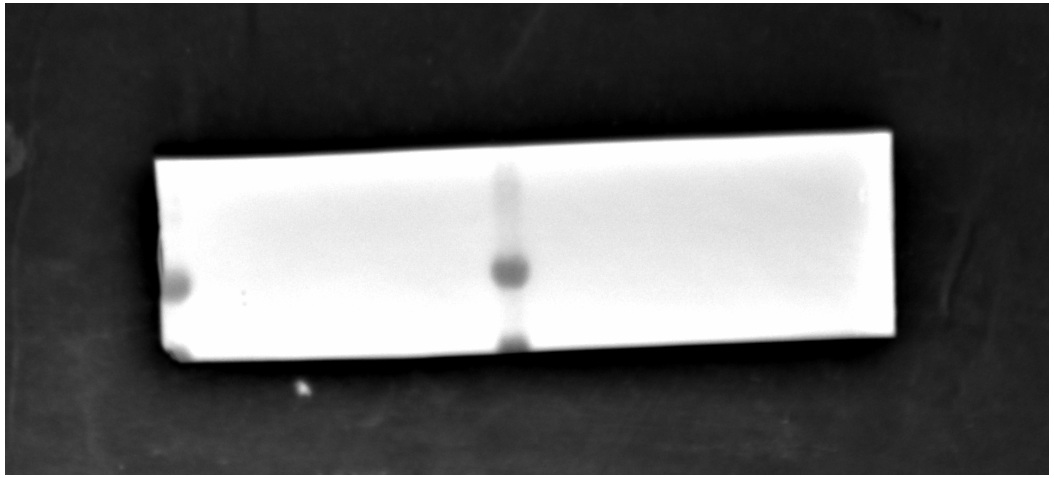

Ponceau

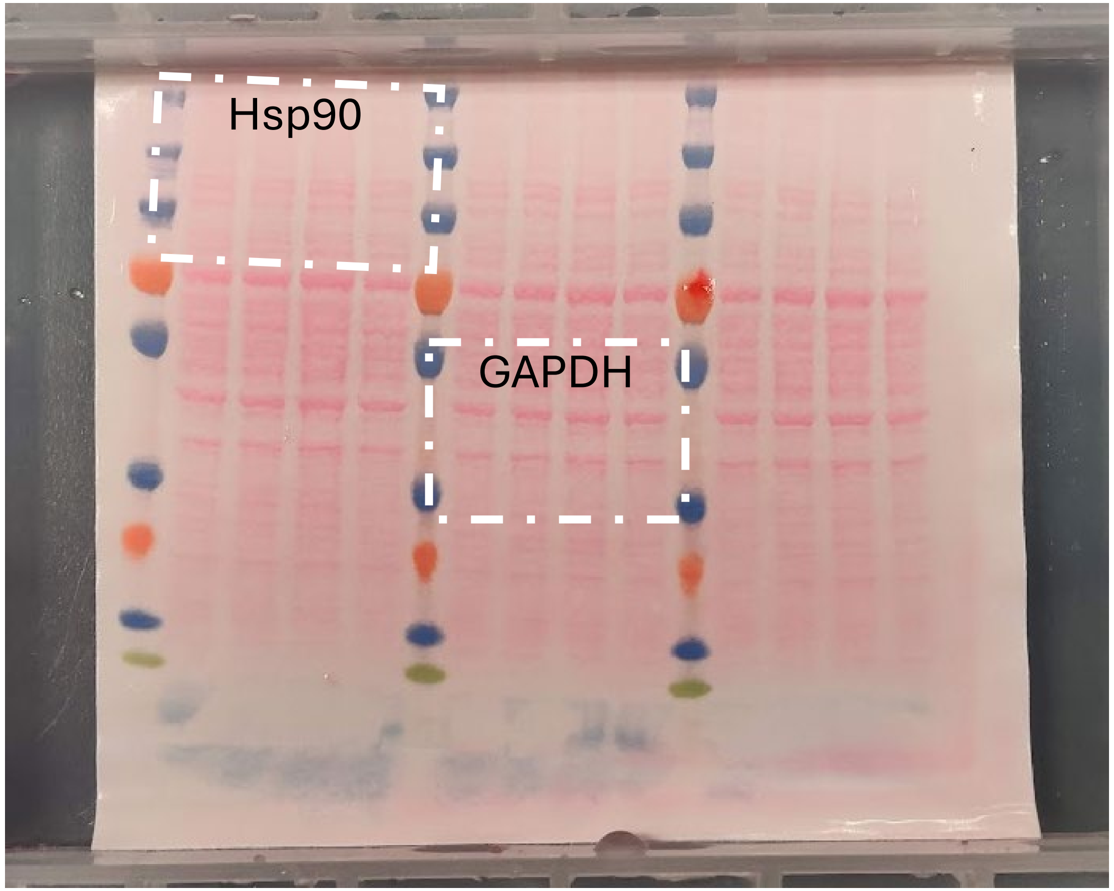

Hsp90

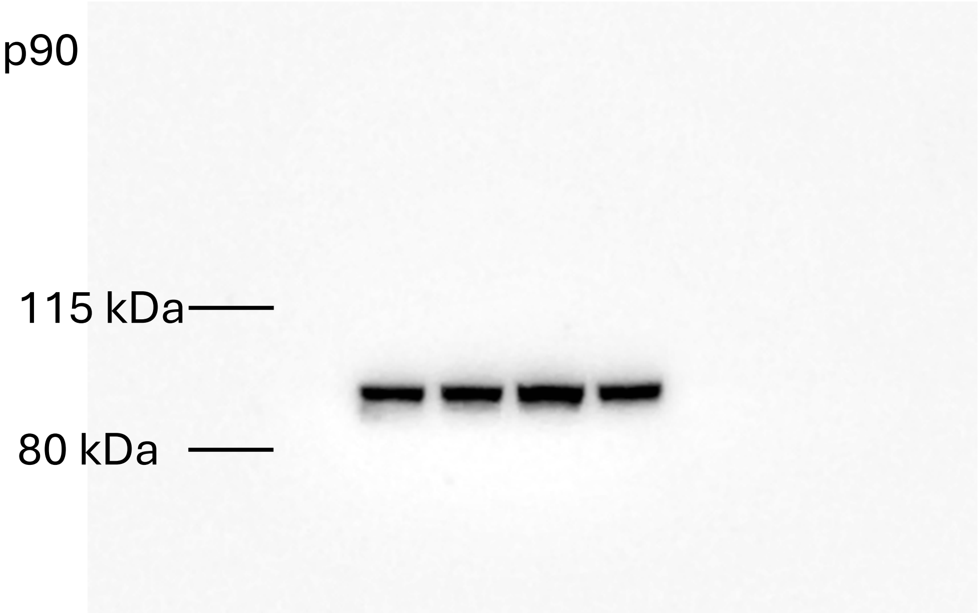

GAPDH

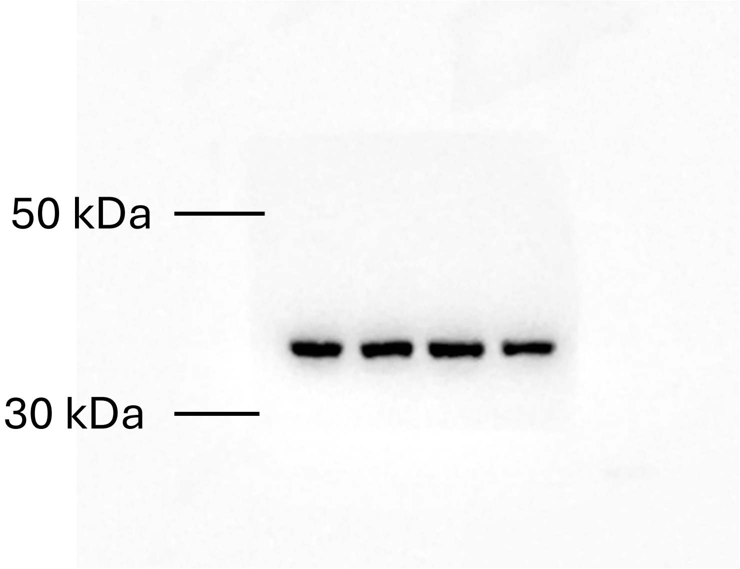

Ponceau

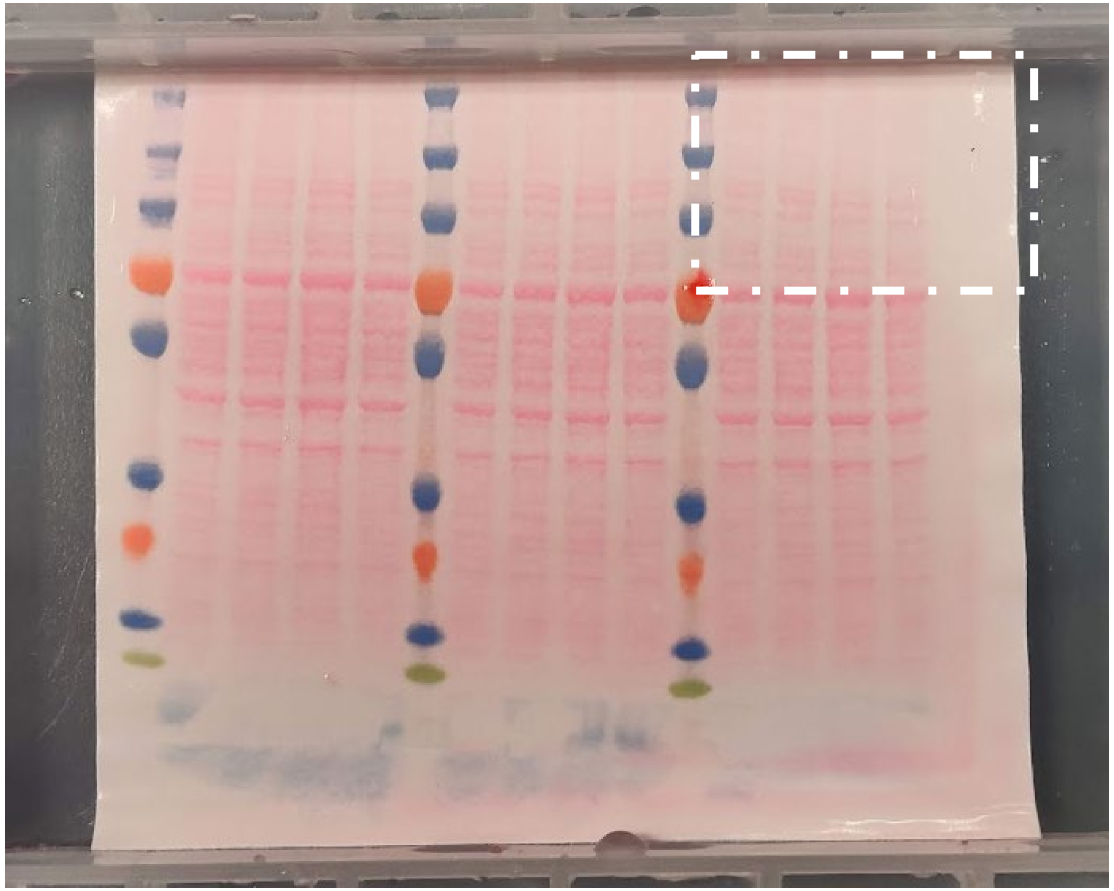

hsp90

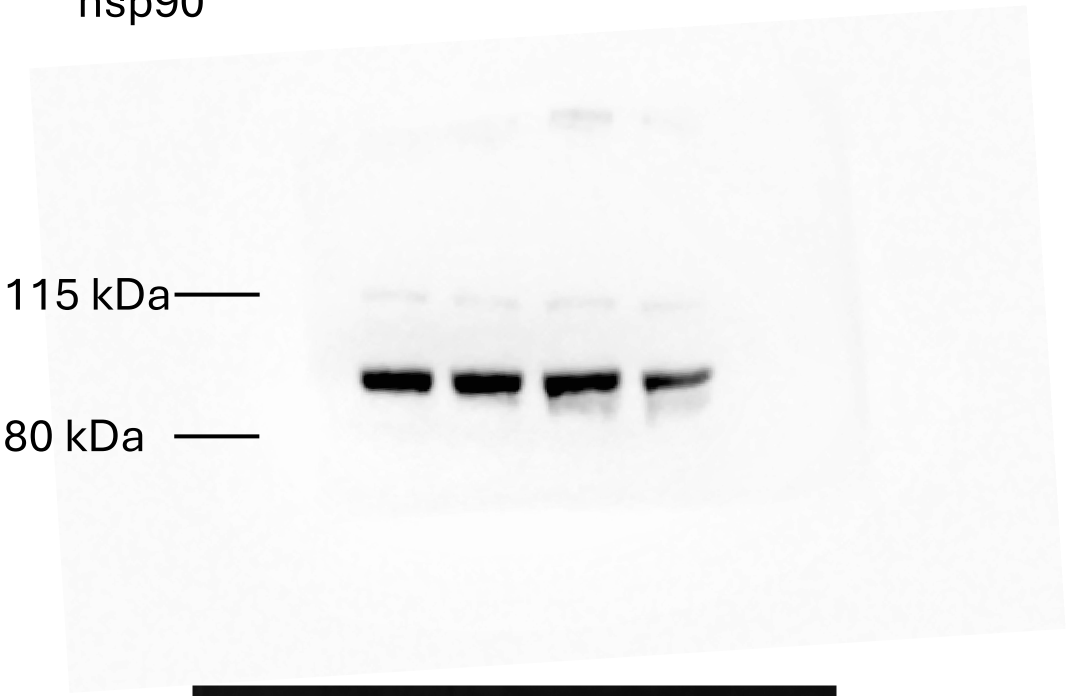

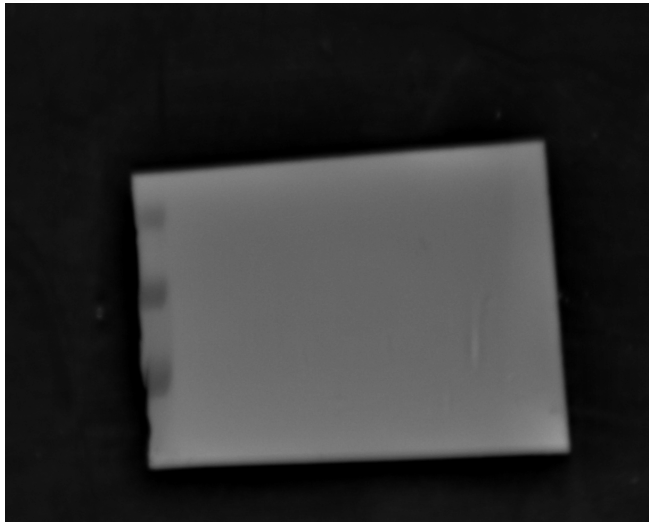

Ponceau

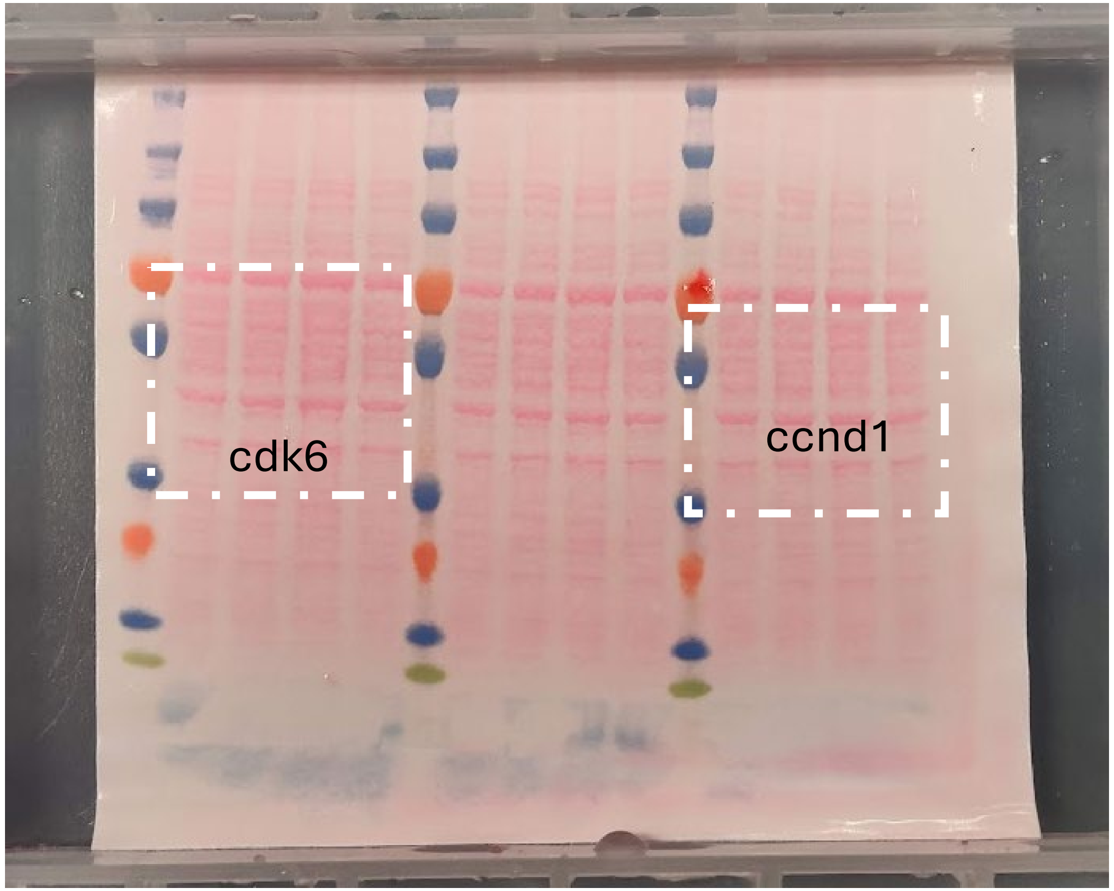

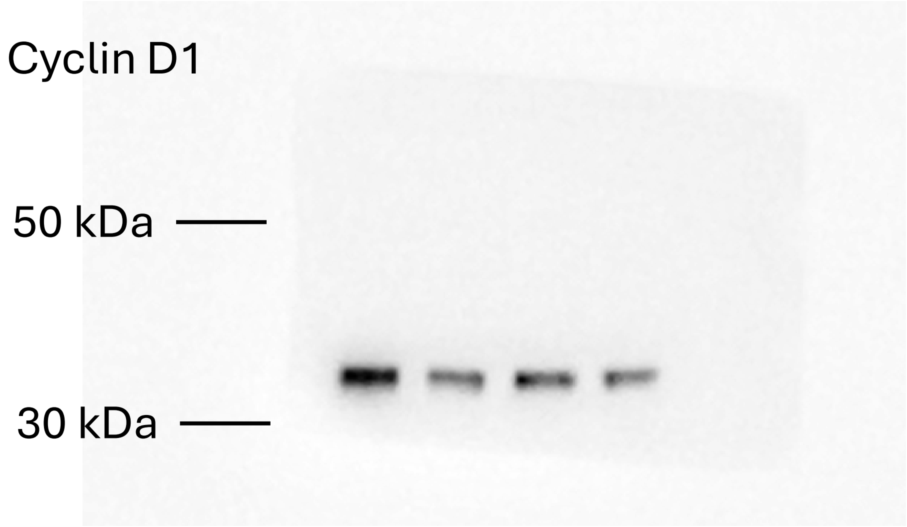

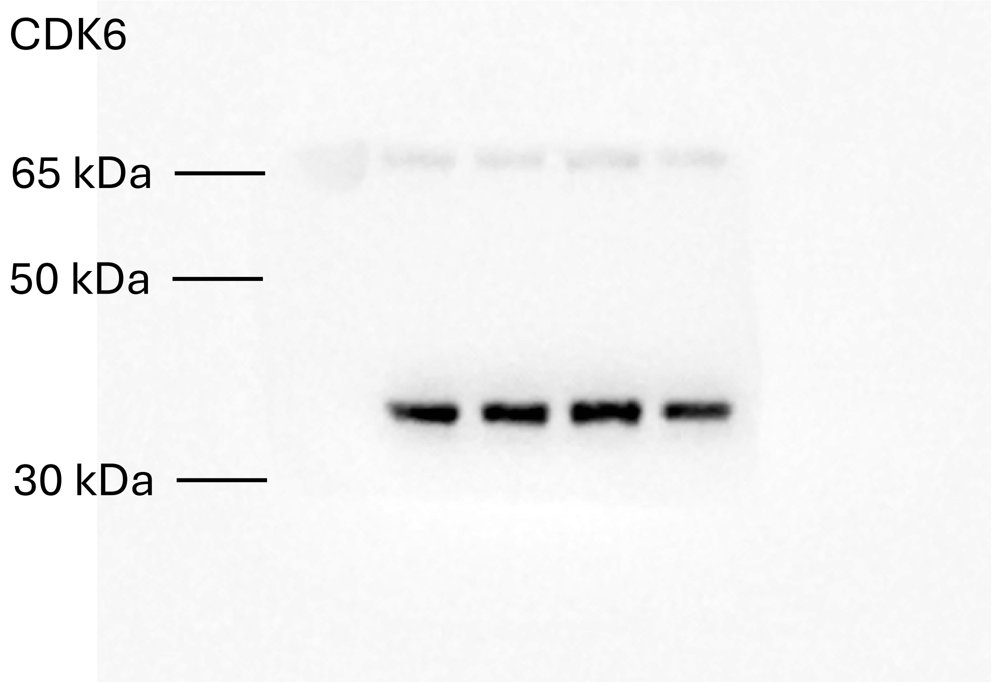

Ponceau

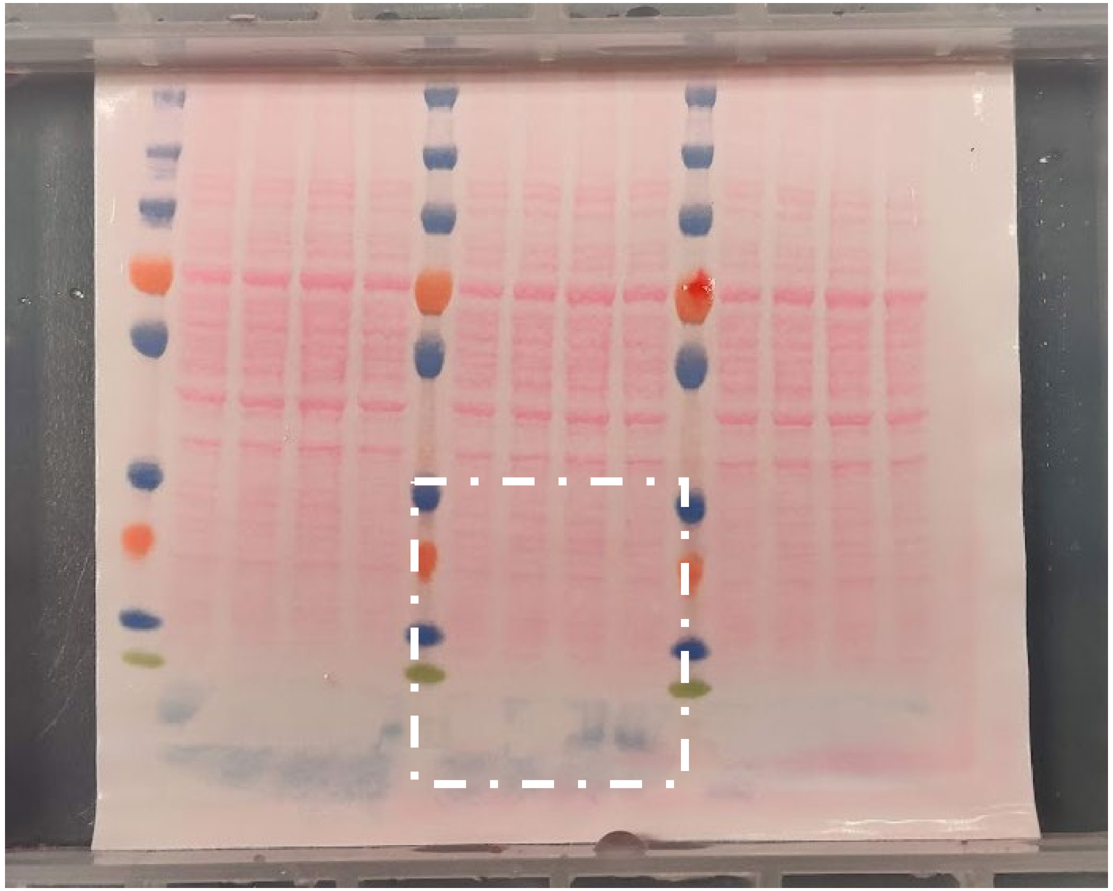

p21

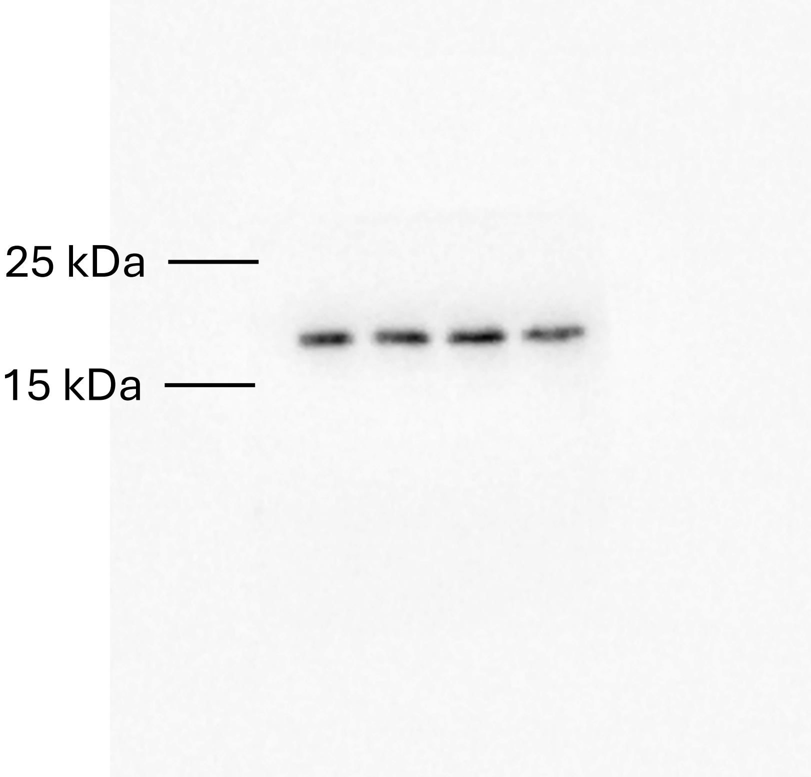

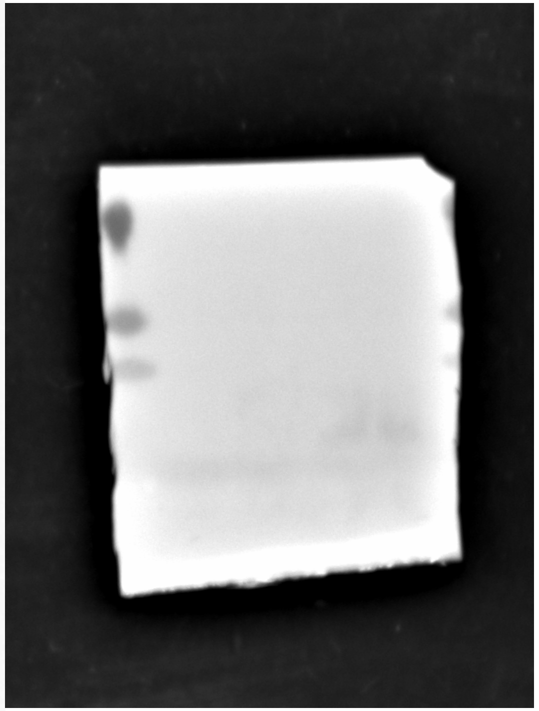

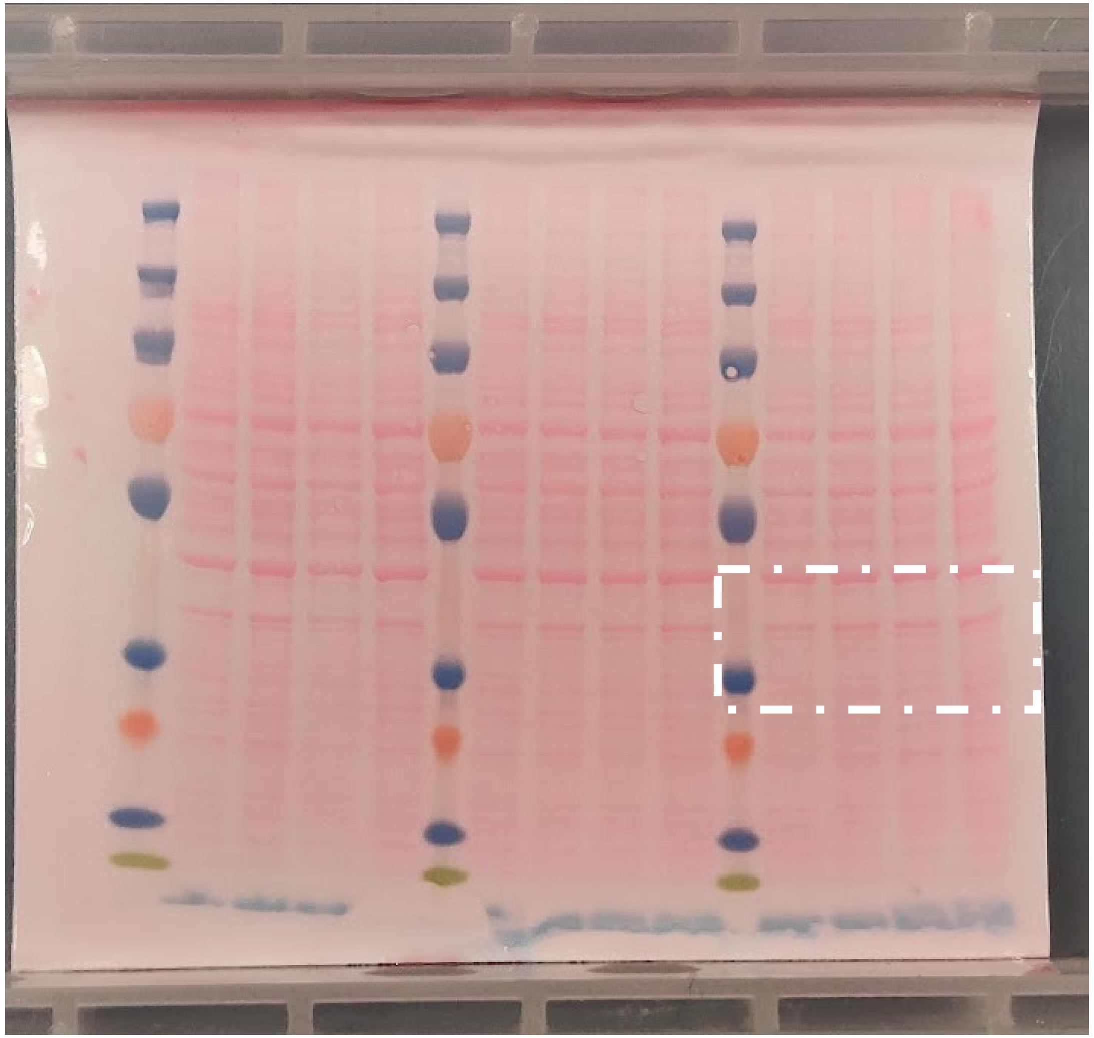

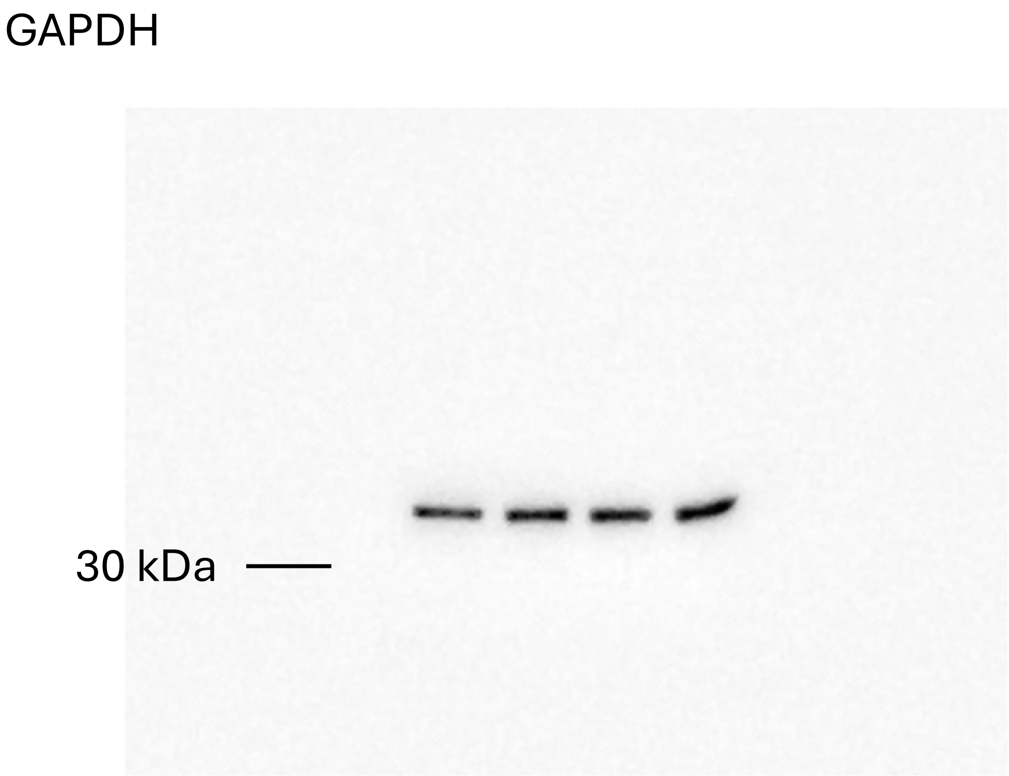
GAPDH
30 kDa

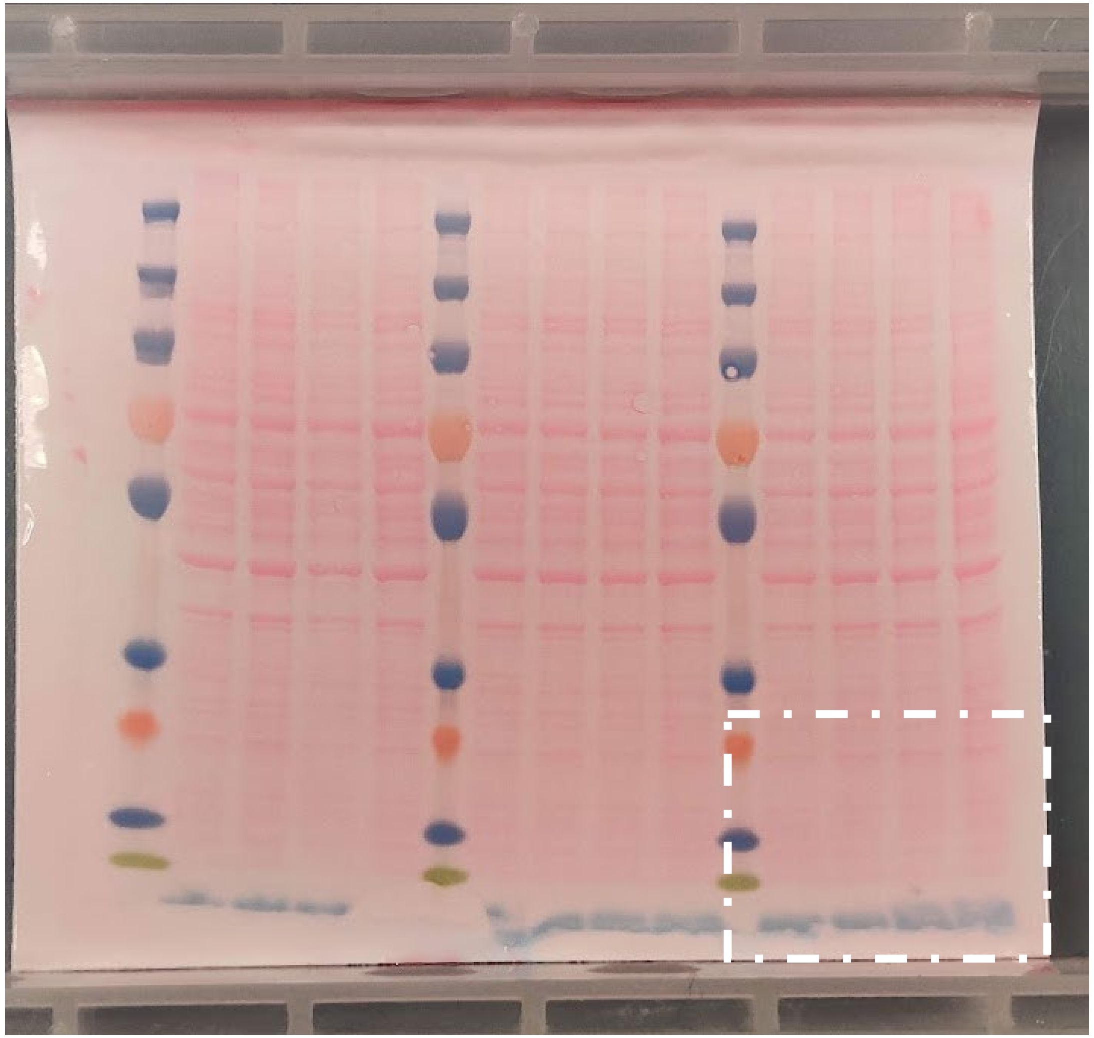

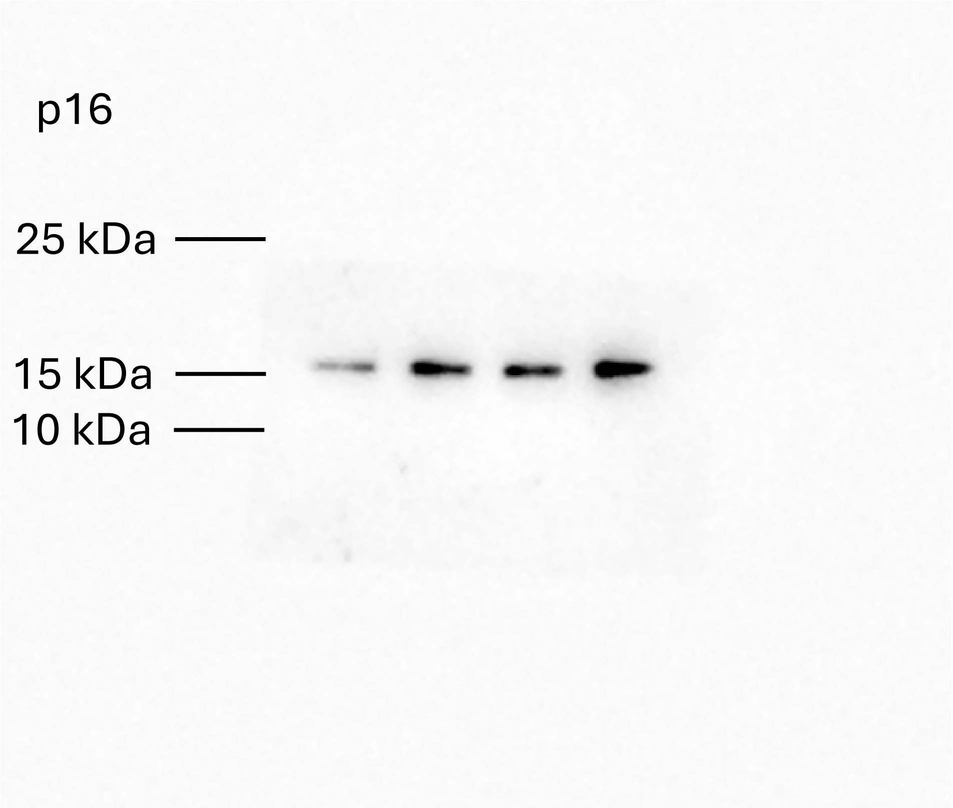
p16
25 kDa
15 kDa
10 kDa
