# Supplementary figures and images for: Targeting senescence induced by age or chemotherapy with a polyphenol-rich natural extract improves longevity and healthspan in mice
Source: Nat Aging. 2024 Jul 1;4(9):1231–48. doi: 10.1038/s43587-024-00663-7 (PMC11408255; doi:10.1038/s43587-024-00663-7)

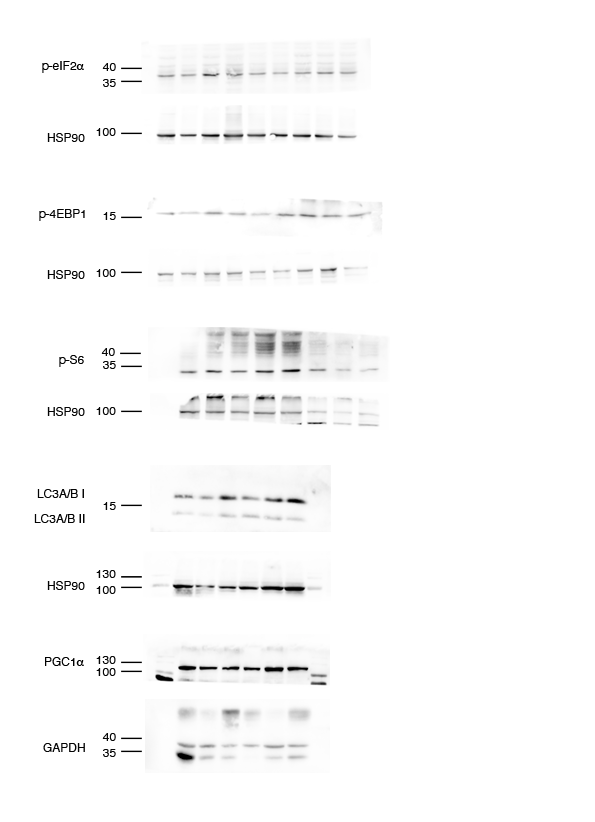

Supplement: Supplementary file 15 — Unprocessed western blots. [file 43587_2024_663_MOESM15_ESM.tif]

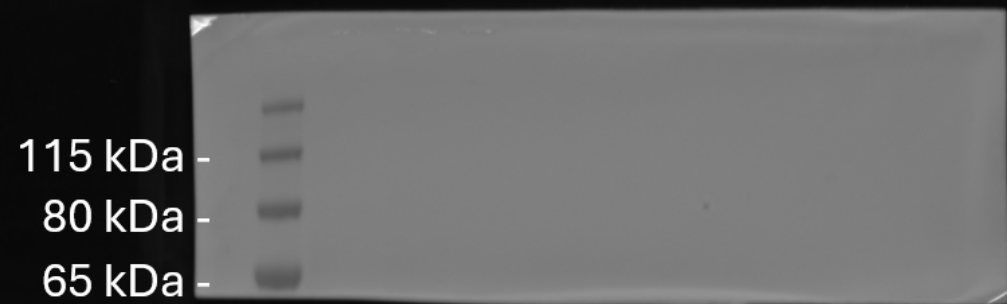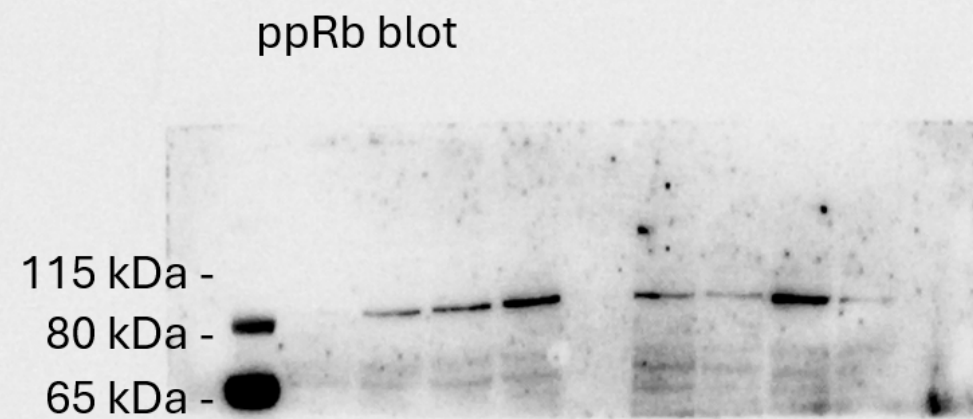

Ponceau Staining

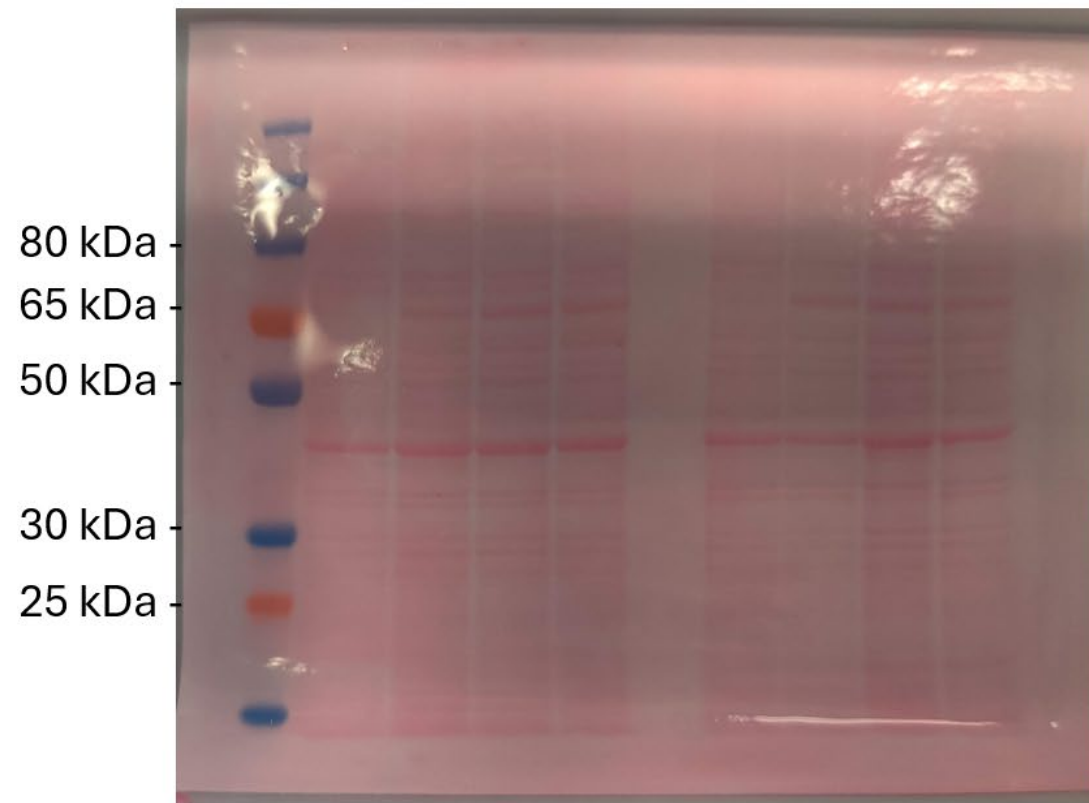

ACTB

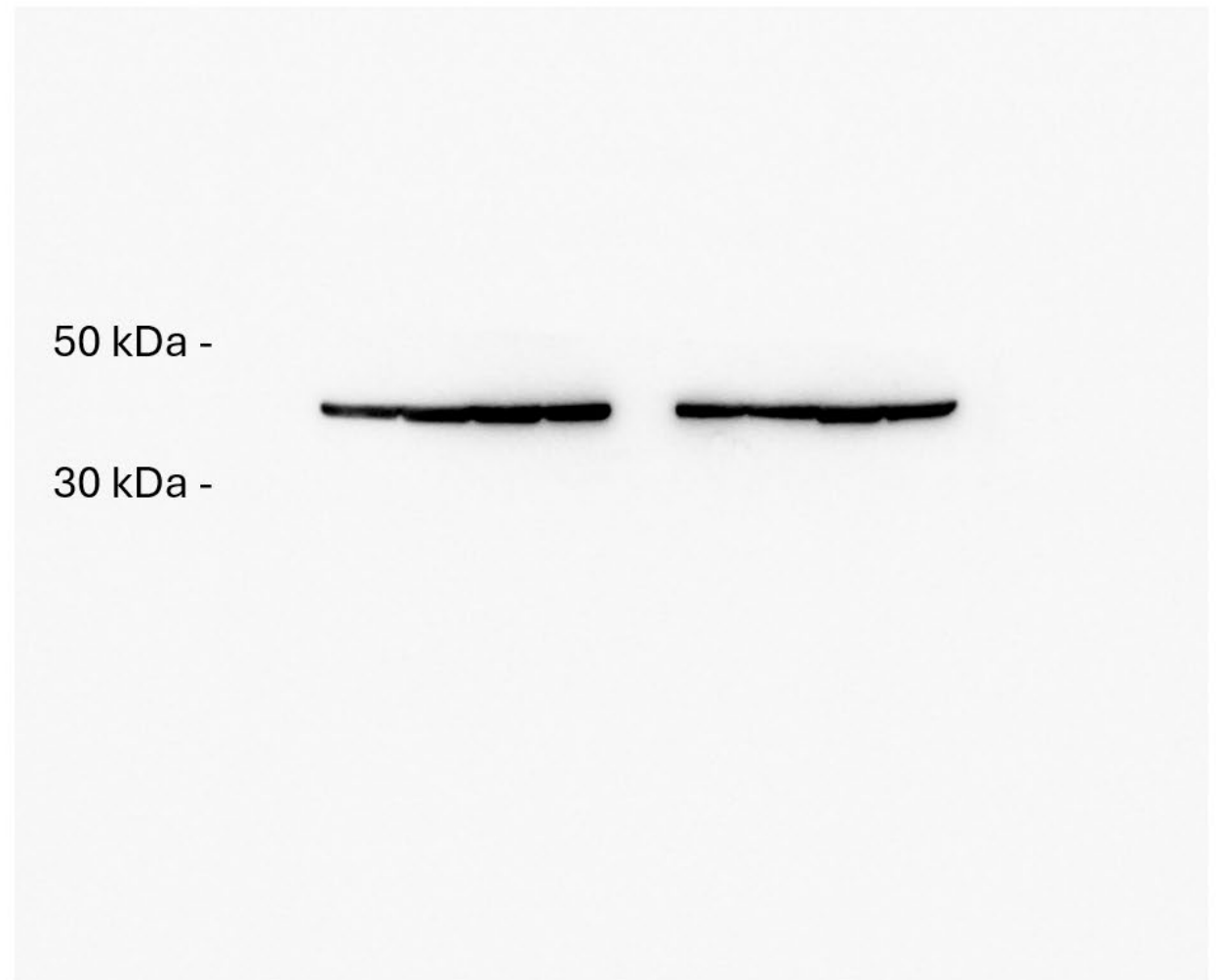

Supplement: Supplementary file 16 — Unprocessed western blots. [file 43587_2024_663_MOESM16_ESM.pdf]
